# Genotype and environment interaction study shows fungal diseases and heat stress are detrimental to spring wheat production in Sweden

**DOI:** 10.1371/journal.pone.0285565

**Published:** 2023-05-10

**Authors:** Ajit Nehe, Ulrika Dyrlund Martinsson, Eva Johansson, Aakash Chawade

**Affiliations:** 1 Department of Plant Breeding, Swedish University of Agricultural Sciences, Alnarp, Sweden; 2 Husshållnigssällskåpet, Borgeby, Sweden; Tocklai Tea Research Institute, INDIA

## Abstract

Spring wheat is an economically important crop for Scandinavia and its cultivation is likely to be affected by climate change. The current study focused on wheat yield in recent years, during which climate change-related yield fluctuations have been more pronounced than previously observed. Here, effects of the environment, together with the genotype and fungicide treatment was evaluated. Spring wheat multi-location trials conducted at five locations between 2016 and 2020 were used to understand effects of the climate and fungicides on wheat yield. The results showed that the environment has a strong effect on grain yield, followed by the genotype effect. Moreover, temperature has a stronger (negative) impact than rainfall on grain yield and crop growing duration. Despite a low rainfall in the South compared to the North, the southern production region (PR) 2 had the highest yield performance, indicating the optimal environment for spring wheat production. The fungicide treatment effect was significant in 2016, 2017 and 2020. Overall, yield reduction due to fungal diseases ranged from 0.98 (2018) to 13.3% (2017) and this reduction was higher with a higher yield. Overall yield reduction due to fungal diseases was greater in the South (8.9%) than the North zone (5.3%). The genotypes with higher tolerance to diseases included G4 (KWS Alderon), G14 (WPB 09SW025-11), and G23 (SW 11360) in 2016; G24 (SW 11360), G25 (Millie), and G19 (SEC 526-07-2) in 2017; and G19 (WPB 13SW976-01), G12 (Levels), and G18 (SW 141011) in 2020. The combined best performing genotypes for disease tolerance and stable and higher yield in different locations were KWS Alderon, SEC 526-07-2, and WPB 13SW976-01 with fungicide treatment and WPB Avonmore, SEC 526-07-2, SW 131323 without fungicide treatment. We conclude that the best performing genotypes could be recommended for Scandinavian climatic conditions with or without fungicide application and that developing heat-tolerant varieties for Scandinavian countries should be prioritized.

## 1. Introduction

Wheat (*Triticum aestivum* L.) is the third most important cultivated food crop globally, contributing around 20% of the protein in the human diet worldwide [1]. In 2019–20 wheat was grown on 239 million hectares and overall global production was 899 million tonnes [2]. In Sweden, wheat is grown on about 15% of cultivated land and is an important part of the agricultural economy [2]. In 2019, total wheat production for Sweden amounted to 3.48 million tonnes from 0.47 million ha of land [2]. Globally, the grain yield of wheat has steadily increased during the last 50 years, although lately there is indications that the wheat yield has plateaued [3]. One of the main challenges for future improvement in wheat production will be increasing resistance to biotic stresses such as diseases, and abiotic stresses such as frequent droughts triggered by climate change. Countries like Sweden, along with other Nordic countries (Finland, Denmark, Iceland, Norway), located from 54° to 69° north latitude, are the most northerly countries to grow field crops and are more threatened by climate change through larger fluctuations and extreme spells [4–6]. In Sweden, depending on disease severity, wheat yield fluctuates and creates market instability and economic losses. Thus, understanding genotype, environment and their interaction have become more important than ever for developing new cultivars that can produce higher and consistent yields across different environments.

The major diseases that affect wheat yield in Nordic conditions are leaf blotch caused by *Septoria tritici*, *Stagonospora nodorum* blotch, tan spot, powdery mildew, leaf stem (upcoming) and strip rust. In Sweden, to avoid yield loss, farmers regularly apply fungicide treatments, which increase production costs and have adverse effects on environmental and human health [7, 8]. In southern Sweden in particular, farmers use fungicide more frequently as relatively high temperatures and humidity result in higher disease pressure [7]. In the Nordic environment, agricultural production is influenced by the characteristic climate of short and intense growing seasons with long days. Traditionally, crop production in this region is regularly affected by early and late frost, but generally experiences fewer plant diseases than elsewhere, mainly because of its cold weather, which is unfavorable for pathogens and pests [9]. The predicted climate change scenario, with increases in temperature could increase humidity and, along with an increase in CO_2_ level in the air, create ideal conditions for diseases to thrive [10]. In Sweden, with a change in climate, it is predicted that the temperature will increase by around 0.5°C by 2050 [11]. Some estimates show that each degree increase in temperature will reduce global wheat production by 6% [12, 13]. One of the reasons for yield reduction will be a change in the dynamics of host-plant interactions due to the introduction of diseases into new areas. If favorable conditions for pathogens prevail, the long-term survival of these diseases could cause problems, along with the arrival of new ones. A recent study on 80 fungi and oomycete crop pathogens in 12 crops, and utilizing climate change models, predicted increased agricultural production at high latitudes although with an increase in disease pressure [14].

For sustainable wheat production, wheat breeders need to understand and develop breeding strategies based on several factors. For example resistant genotypes, genotype behavior in multi-environments, optimal production regions and weather pattern changes. Screening crop germplasms for resistance to fungal disease, along with appropriate management systems, will help with achieving stable yield production. Breeding new varieties for higher yield, resistance to disease, wider adaptability, and stable production across different regions has traditionally been the goal of plant breeding all around the world [15]. There are limited recent studies in the Nordic region on cultivated crops in which the genotype × environment interaction (GEI) has been studied in terms of fungal diseases and changes in weather conditions [16, 17]. The aim of the present study to evaluate GEI over five years from 2016 to 2020 using a set of wheat genotypes across five agricultural production regions with differing environmental conditions, under two contrasting fungicide treatments, that is Untreated (FUT) and Treated (FT). Our objective was to identify the major environmental factors that affect wheat yield, resistant genotypes for fungal diseases, stable genotypes across the locations and the best wheat-growing region.

## 2. Materials and methods

### 2.1 Experimental locations and growing conditions

**Fig 1:**
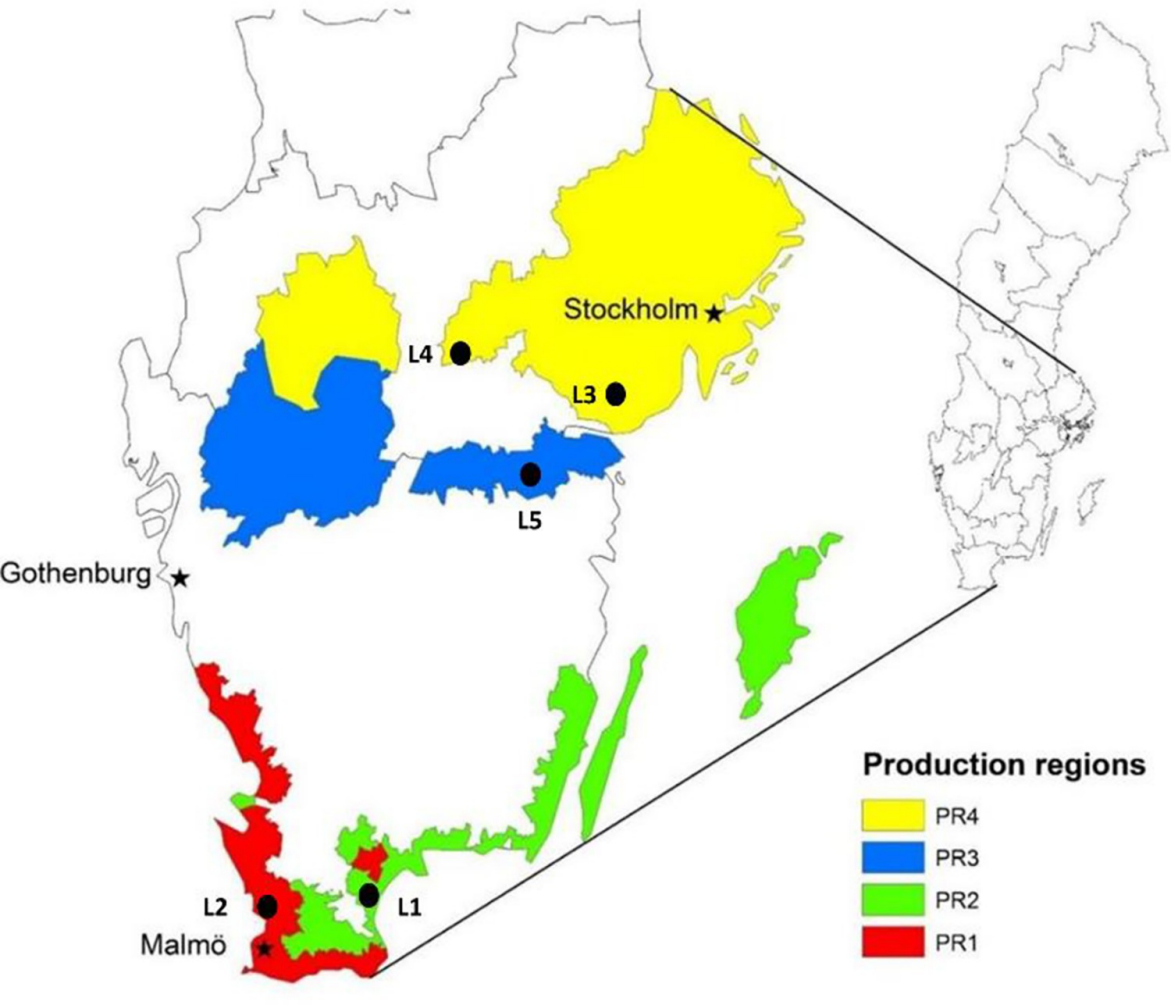
Map showing the four most productive agricultural regions and experimental locations in Sweden. (Adapted from Lantz, Prade [18]).

This study utilized five years (2016 to 2020) of historical data from Swedish national trials (multi-location trials conducted by Hushållningssällskapet) at five locations in agricultural production regions, in either the South or North zone of Sweden (Fig 1). All data from these field trials are available on the website https://sverigeforsoken.se/s. During the five years utilized, significant climate change-related weather fluctuations and severe effects on grain yield were present. This was in particular evident for the crop yield in 2018, which was similar to the rest of North Europe historically low. Sweden is a country that is 1574 km long, from latitude 55° N (Smygehuk) to 69° N (Treriksröset), with the most southerly parts corresponding to Southern Alaska. However, due to its proximity to the warm Gulf Stream in the Atlantic Ocean, the winters are much milder than in similar latitudes for example in Siberia and Canada. Climate conditions vary from temperate in the south to polar in the north. Most of the agricultural production regions are located in the southern provinces and central plain regions, along coasts, lakes and rivers. The selected experimental locations (L) were representative of four agricultural production regions of Sweden, contributing to more than 90% of the cultivable land in Sweden. L1 and L2 represent the South zone, and L3, L4 and L5 represent the North zone within the agricultural production region (Fig 1). These locations have an annual rainfall of around 500 mm and receive precipitation all year round. Spring wheat is generally sown in the spring around mid-April and harvested around mid-August. Information about the previous crop before spring wheat and the sowing date at each location is included in S1 Table. Agronomic data, such as the date of sowing and harvesting, were recorded. Crop duration is the time from sowing to harvesting. Weather data such as ambient temperature and rainfall were collected from the nearest weather station. Average temperature and rainfall during the crop growth period were used in data analysis. Thermal time was calculated by summing the average minimum and maximum daily temperature over the crop growing duration, with a base temperature of zero.

### 2.2 Plant material and experimental design

In each year, as determined by the performing actor of the trial (Hushållningssällskapet), a specific set of genotypes, between 19 and 26, were sown across all locations. This set of genotypes was used as the study was originally focused on analyzing the location effect in individual years along with local farmers’ practices of controlling fungal diseases. The date of sowing and harvesting and the list of genotypes are shown in S1 and S2 Tables. The criteria for genotype selection were to include genotypes that are advanced breeding lines having high yield potential, commercially important to the Swedish economy, and contributing to total wheat production. Two contrasting treatments applied in this experiment were fungicide untreated (FUT) and fungicide treated (FT). To control fungal diseases, commonly adopted practices and fungicides used by farmers were included. In this study, fungicide was applied in such a way that there was no significant reduction in yield due to any fungal diseases the with FT treatment. The most common fungicides used for application to wheat during the time of trials are Tilt 250 EC, Proline EC 250, and Comet Pro according to disease severity at each location and year. Other crop management practices such as fertilizers, insecticides, and herbicides were applied as in accordance with common practices in each location.

The trials were conducted in a split-plot experimental design, with two replicates in each treatment across all the locations and years. Genotypes were randomized in each replicate. Plot sizes were different at different locations, with four rows and an average plot size of 15m^2^. The distance between rows was 20 cm, and 400 seeds were sown per square meter. At all locations, soil moisture was available only from precipitation during the growing season, and no supplementary irrigation was provided. The crop was harvested at maturity using a combine harvester. Grain yield data were collected after threshing and drying to a moisture level of 80%.

### 2.3 Statistical analysis

Analysis of variance (ANOVA) procedures for a split-plot design were used to analyze treatment (FUT and FT) and genotype effects and test their interaction with locations. We used GenStat version 22 (www.genstat.com; VSN International Ltd, Hemel Hempsted, UK), with replicates treated as random effects and genotypes as fixed effects (Table 1). In the Split-plot analysis, treatment was considered to be the main treatment and genotype the sub-treatment. A cross-location ANOVA was applied to analyze fungicide treatment and genotype effects across locations and the interaction with locations, considering fungicide treatments and genotypes to be fixed effects and replicates and locations to be random effects. In the ANOVA results presented only the main effect and associated interactions are shown. The least significant difference values (LSD) along with P-values are presented in the ANOVA table for better comparison between different variables. Disease tolerance traits were calculated as percent yield reduction for FUT compared to FT treatment. Percent yield reduction for FUT wheat in relation to FT treatment is used as a disease tolerance indicator, because less reduction in yield under FUT treatment indicates resistance of the genotype to fungal diseases. To study G × E for yield and disease tolerance, AMMI analysis was conducted using GenStat version 21. AMMI ANOVA for disease tolerance is shown in Table 2. For GY, “Who won where/what” and AMMI 1 Bi-plots were produced using GEA-R (https://data.cimmyt.org) to identify the best genotypes and location and to rank the disease tolerance of genotypes. To reveal the genotypes’ combined performance for higher GY, yield stability across the locations, and resistance to diseases, we used ranking by summing the individual trait-ranking of these three parameters. Pearson’s correlation coefficient and linear regressions were calculated to quantify associations between different environmental parameters and average grain yield for each location using GenStat version 21. The key weather parameters used were average temperature (°C), average rainfall (mm), and crop duration (days from sowing to harvesting at physiological maturity) at each location. Regression coefficients are presented for all variables for the linear regressions. The standard deviation is represented by the error bars in the histograms.

**Table 1:** Minimum (Min), maximum (Max), and mean values of wheat grain yield (t ha^-1^) under fungicide untreated (FUT) and fungicide treated (FT) treatment along with percent yield reduction for FUT as compared to FT for the years 2016 to 2020, and analyses of variance (ANOVA) for effects of genotypes, year, location and their interactions on grain yield.

		2016				2017				2018				2019				2020			
	Treatment		Min	Max	Mean		Min	Max	Mean		Min	Max	Mean		Min	Max	Mean		Min	Max	Mean
**L1**	FUT		6.30	9.50	**8.06**		5.47	10.0	**8.84**		3.63	5.30	**4.59**		-	-	**-**		8.14	10.4	**9.46**
	FT		6.54	9.46	**8.22**		7.97	11.3	**10.25**		3.67	5.78	**4.60**		-	-	**-**		8.98	11.2	**10.3**
	%red		-5.12	8.05	**1.90**		-2.98	31.9	**13.74**		-16.54	11.4	**0.28**		-	-	**-**		2.87	18.0	**8.47**
**L2**	FUT		7.46	10.8	**9.42**		4.03	9.53	**7.52**		4.01	6.43	**5.71**		7.88	10.85	**9.07**		5.94	9.13	**7.89**
	FT		8.68	11.9	**10.7**		6.48	11.49	**10.5**		4.12	6.23	**5.72**		8.49	10.47	**9.35**		6.40	9.47	**8.42**
	%red		2.78	23.5	**11.7**		17.1	37.81	**28.6**		-16.67	27.6	**0.17**		-7.20	11.72	**2.96**		-4.37	14.5	**6.20**
**L3**	FUT		7.62	10.4	**8.96**		8.53	11.57	**10.4**		-	-	**-**		7.22	9.08	**8.20**		9.28	11.7	**10.5**
	FT		8.15	11.0	**9.77**		9.11	12.32	**11.2**		-	-	**-**		6.64	9.46	**8.21**		9.57	12.6	**11.4**
	%red		1.20	18.4	**8.19**		0.27	12.71	**6.72**		-	-	**-**		-17.8	15.54	**-0.42**		1.46	14.9	**8.15**
**L4**	FUT		7.29	12.5	**9.89**		6.87	12.62	**11.0**		4.54	8.73	**7.31**		8.41	11.7	**10.7**		-	-	**-**
	FT		8.90	13.6	**11.5**		8.78	13.97	**12.3**		5.11	8.79	**7.46**		9.34	12.6	**11.4**		-	-	**-**
	%red		3.36	23.0	**14.1**		0.00	21.75	**10.3**		-8.37	12.5	**2.04**		-1.60	13.3	**6.06**		-	-	**-**
**L5**	FUT		5.19	8.63	**6.46**		8.90	14.63	**12.1**		8.04	11.0	**10.0**		8.82	10.9	**10.3**		11.1	13.3	**12.1**
	FT		4.85	8.46	**7.04**		10.7	16.29	**13.3**		8.15	11.5	**10.3**		8.90	11.5	**10.4**		11.4	13.6	**12.5**
	%red		-10.4	19.1	**8.10**		-1.88	23.30	**9.05**		-1.78	9.32	**3.17**		-3.70	5.47	**0.96**		1.12	6.09	**3.10**
**Mean**	FUT		6.85	10.2	**8.56**		7.19	10.89	**9.98**		5.06	7.87	**6.90**		8.12	10.3	**9.55**		8.61	11.1	**9.99**
	FT		7.42	10.5	**9.44**		9.39	12.72	**11.5**		5.26	8.08	**6.95**		8.56	10.5	**9.83**		9.09	11.7	**10.7**
	%red		2.11	16.2	**9.41**		6.52	24.18	**13.3**		-10.8	15.2	**-0.22**		-3.96	8.08	**2.75**		0.27	13.4	**6.48**
		**Df**	**%TSS**	**LSD**	**P-Val**	**Df**	**%TSS**	**LSD**	**P-Val**	**DF**	**%TSS**	**LSD**	**P-Val**	**DF**	**%TSS**	**LSD**	**P-Val**	**Df**	**%TSS**	**LSD**	**P-Val**
**G**		24	16.9	0.27	***	25	15.8	0.28	***	25	6.59	0.24	***	19	11.33	0.37	***	18	8.56	0.28	***
**T**		1	5.82	0.54	**	1	15.6	0.40	***	1	0.01	0.08	0.74	1	0.99	0.37	0.11	1	3.90	0.21	**
**L**		4	59.1	1.78	**	4	48.9	0.60	***	3	89.1	0.63	***	3	61.38	1.21	**	3	76.5	1.00	**
**T x G**		24	0.66	0.60	***	25	3.83	0.52	***	25	0.22	0.69	0.05	19	0.90	0.57	0.40	18	0.35	1.12	0.19
**G x L**		96	3.11	1.78	***	100	9.12	0.79	***	75	1.84	0.62	***	57	8.32	1.25	***	54	4.15	1.12	***

Significant at 5%

*1%

** and 0.1%

*** level. (G: Genotype, T: Treatment, L: Location, %TSS: percent total sum of square).

**Table 2:** Showing AMMI ANOVA for disease tolerance and different effects of genotype (G), treatment (T), location (L), and interaction of genotype × location (G × L) for five separate years (2016 to 2020).

	2016					2017					2018					2019					2020				
Source	d.f.	s.s.	m.s.	P-val	%TSS	d.f.	s.s.	m.s.	P-val	%TSS	d.f.	s.s.	m.s.	P-val	%TSS	d.f.	s.s.	m.s.	P-val	%TSS	d.f.	s.s.	m.s.	P-val	%TSS
T	124	10712	86	***	44.4	129	23116	179.2	***	80.1	129	15869	123.0	**	29.8	79	7941	100.5	NS	50.6	94	5637	60.0	NS	52.9
G	24	2514	104	***	10.4	25	3423	136.9	***	11.9	25	2754	110.2	NS	5.2	19	2150	113.1	NS	13.7	18	798	44.3	NS	7.5
L	4	12243	2651	***	50.7	4	18078	4408.6	***	62.7	3	28985	5886.2	***	54.4	4	1942	570.9	*	12.4	3	2950	696.9	**	27.7
G x L	96	4145	43	NS		100	3830	38.3	*		100	11333	113.3	*		57	4767	83.6	NS		72	2701	37.5	NS	
Total	249	24144	97			259	28854	111.4			259	53301	205.8			159	15683	98.6			189	10651	56.4		
IPCA 1	27	1832	67	*	44.1	28	1502	53.6	**	39.2	28	7565	270.2	***	66.8	21	3407	162.3	*	71.5	21	1612	76.8	*	59.7
IPCA 2	25	1374	55	NS	33.1	26	1074	41.3	NS	28.0	26	2134	82.1	NS	18.8	19	993	52.3	NS	20.8	19	696	36.6	NS	25.8
Residuals	44	939	21	NS		46	1255	27.3	NS		46	1634	35.5	NS		17	366	21.5	NS		32	393	12.3	NS	
Error	120	5241	43			125	3522	28.2			125	10229	81.8			76	6824	89.8			90	4201	46.7		

Significant at 5%

*, 1%

** and 0.1%

*** level.

## 3. Results

### 3.1 Growing conditions and their effect on grain yield

Background data including dates of sowing and harvesting, mean crop duration, temperature, thermal time, and rainfall for each location and year are given in S1 Table. These data showed that, 2018 was the hottest (17.5°C) year among the five years evaluated, with the lowest mean rainfall (129 mm) and shortest crop duration (108 DAS), whereas 2017 was the coldest (13°C) year with the longest crop duration (155 DAS). However, the thermal time of the crop growing period across all years and locations does not show much variation and ranged from 1990 (2020) to 2067 (2016)°CD. Comparing temperature and rainfall in individual locations and years, in 2018, location L4 had the highest (18.0°C) mean temperature with the shortest crop duration (99 DAS) and L5 had the lowest rainfall (87.8 mm).

**Fig 2:**
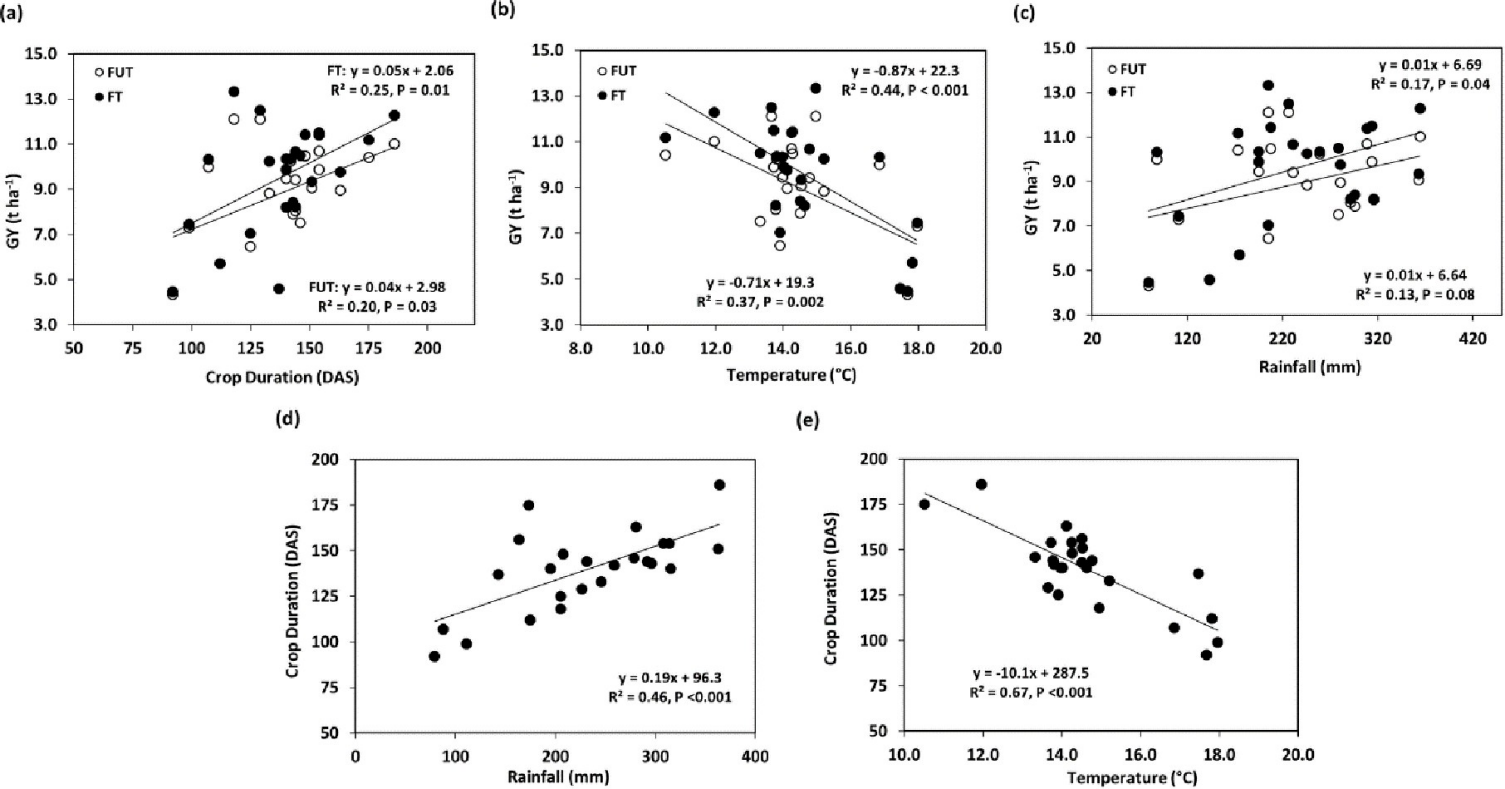
Linear regression of grain yield (GY, t ha^-1^) with (a) crop duration (DAS) (b) temperature (°C), and (c) rainfall (mm), and crop duration (DAS) with (d) temperature (°C) and (e) rainfall (mm). (Data are the means for each location in each year).

Considering the effect of the environment on grain yield (GY) across the locations and years, there was a negative association between GY and temperature (FUT: R^2^ = 0.44, < = 0.001 and FT: R^2^ = 0.37, P = 0.002) under both treatments, whilst there was no association between GY and rainfall under either treatment (Fig 2B and 2C). There was also a positive association between GY and crop duration under both treatments (FUT: R^2^ = 0.20; P = 0.03 and FT: R^2^ = 0.25; P = 0.01) (Fig 2A). Moreover, crop duration was also affected negatively by temperature (R^2^ = 0.67; P <0.001) and positively by rainfall (R^2^ = 0.46; P <0.001) (Fig 2). Similar to the effect of temperature on GY, temperature also affected crop duration more than rainfall with higher temperature related to earlier crop maturity (Fig 2D and 2E).

### 3.2 Impact of agricultural production zones and regions along with fungicide treatment on grain yield

**Fig 3:**
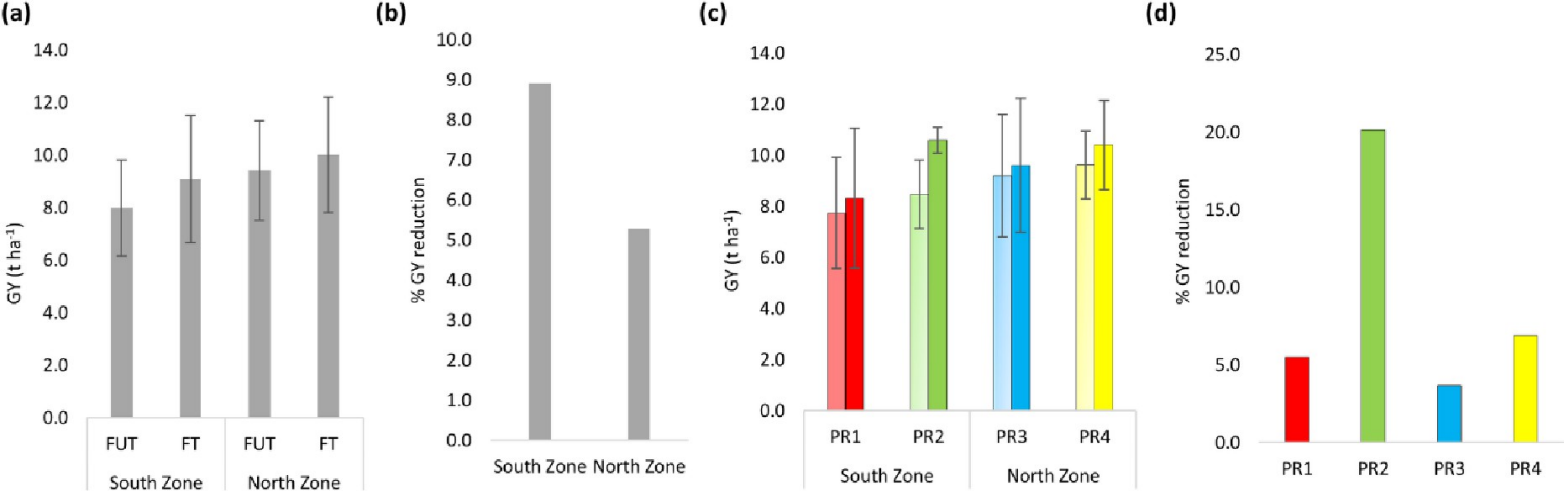
Shows (a) Grain yield (GY, t ha^-1^) under fungicide untreated (FUT) and treated (FT) treatment in the South vs North zone, (b) percent GY reduction under FUT treatment in the South vs North zone, (c) GY (t ha^-1^) under FUT (light colors) and FT (dark colors) treatment and (d) percent grain yield reduction under FUT treatment for four agricultural production regions of Sweden. (Data are means for the period 2016 to 2020 and different colors indicate different production regions similar to Fig 1; error bars represent standard deviation).

Comparing mean grain yield (GY) across years and locations, there was no clear difference between the South and North zone, (Fig 3A). However, despite relatively low rainfall in the South (S1 Table), PR2 (Production Region 2), which comes under the South zone was the most productive region for spring wheat under FT treatment (Fig 3C). PR2 was also the region where the relative yield reduction under FUT treatment compared to FT was higher (20.2%) than in the other regions (Fig 3C and 3D). Overall yield reduction due to fungal diseases was greater in the South (8.9%) than the North zone (5.3%) (Fig 3B).

### 3.3 Grain yield across years, genotype and locations

ANOVAs for the split-plot experimental design with the variables genotype, treatment, location, and their interactions are shown in Table 1 for each year. Across all years, there was no significant difference between the replicates. Overall, most of the effects and interactions were significant except for the effect of fungicide treatment (T) in 2018 and 2019 and the T × G interaction in 2019 and 2020. Comparing all years, the location percent total sum of squares (% TSS) effect was highest, contributing on average 66.6% TSS, followed by the genotype effect with 11.5% TSS, indicating a strong influence of environment on total variation in GY. The strongest location effect was in 2018 (89.1% TSS) (Table 1).

**Fig 4:**
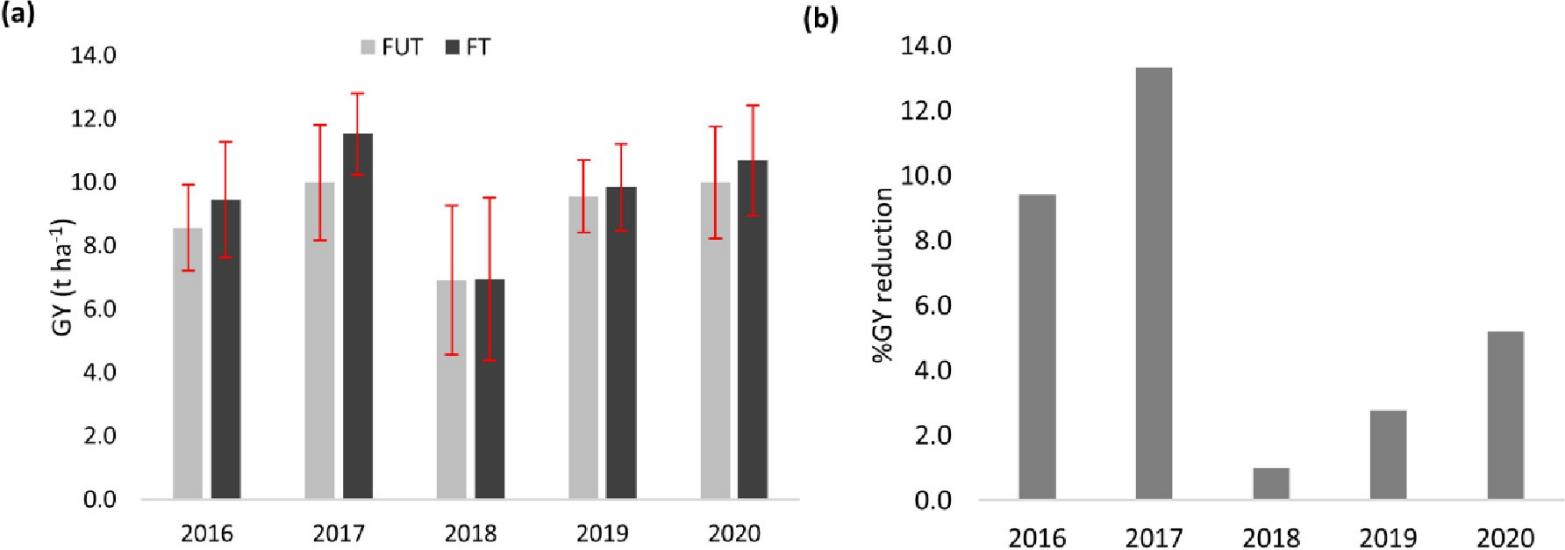
Shows grain yield (GY t ha^-1^) under fungicide untreated (FUT) and fungicide treated (FT) treatment and % relative GY reduction under FUT treatment for each year for spring wheat (Data are means across five locations for each year and the error bar indicates standard deviation).

Over the five years, overall relative GY was highest in 2017 (FUT: 9.98 and FT: 11.5 t ha^-1^) and lowest in 2018 (FUT: 6.90 and FT: 6.95 t ha^-1^) for both treatments. Comparing the overall yield reduction under FUT to the FT treatment, grain yield reduction was 6.07%, with the highest reduction in 2017 (-13.3%) and the lowest in 2018 (-0.22%) (Table 1, Fig 4).

### 3.4 Genotype and environmental effects on grain yield

In the present study, locations were used as the environmental factor to evaluate environmental interactions, due to the fact that the utilized genotype set differed over years but was the same across locations for each year. Thus, genotype and environmental effects for each separate year is presented below.

The four common genotypes were WPB Skye, Happy (SW 91003), Diskett SW 45456 and Quarna. Overall, their relative ranking was consistent across all the years, as in the above sequence except in 2017 when Happy ranked higher than WPB Skye (S2 Table).

For individual years genotype × environment (G × E) interactions are presented below.

#### 3.4.1 G × E 2016

There was significant phenotypic variation between the genotypes with respect to GY (P <0.001, Table 1). Average GY across the genotypes and locations varied from 6.85 to 10.2 t ha^-1^ with a mean of 8.56 t ha^-1^ under FUT and 7.42 to 10.5 t ha^-1^ with a mean GY of 9.44 t ha^-1^ under FT treatment (P <0.001, Table 1).

To determine which genotypes performed better than the mean in combination with a suitable location, an AMMI 1 analysis was performed. In this analysis, the top three high-yielding genotypes under the FUT and FT treatments were G4, G14 and G25 and G25, G13 and G4, respectively (S1A and S1B Fig). Genotypes that showed the combined best performance for GY, stability and resistance were G19, G8 and G14 and G4, G19 and G20 under the FUT and FT treatments, respectively (S2 Table). The locations where the wheat performed better than the overall mean under FUT were L4 and L1, and under FT were L3, L4 and L3. However, the wheat performance at L5 and L2 for the FUT treatment and L2 and L5 for the FT treatment were below the overall mean (S1A and S1B Fig).

**Fig 5:**
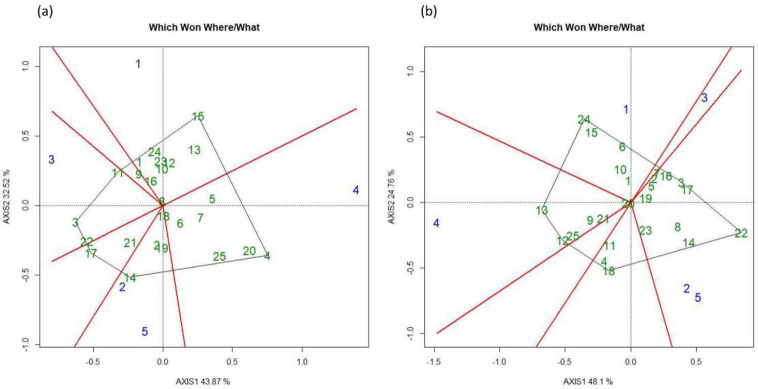
Showing GGE bi-plots for five locations under for (a) fungicide untreated (FUT) and (b) treated (FT) treatments for wheat grain yield in 2016.

The GGE biplot divided genotypes according to the environment where they performed best to achieve higher GY. The GGE Biplot for FUT divided the different locations into six groups. L4 was the most diverted environment whereas L1 and L3 were comparatively less diverse whereas L2 and L5 were relatively similar and grouped closed to each other. The genotypes that grouped at the center and exhibited the most stable performance in all the locations were G8 (WPB Skye), G18 (WPB Scotch) and G6 (WPB Oryx) (Fig 5A). An overall similar trend was also observed under FT treatment. The GGE biplot divided genotypes and locations into six groups. L4 and L1 were the most diverse environment whereas L2 and L5 were comparatively less diverted and grouped close to each other. The genotypes with the most stable performance were G20 (Skandus), G19 (WPB Avonmore) and G1 (Diskett SW 45456) (Fig 5B).

#### 3.4.2 G × E 2017

Overall in 2017, there was highly significant phenotypic variation between the genotypes with respect to GY (P <0.001, Table 1). The average GY across the genotypes and locations varied from 7.19 to 10.9 t ha^-1^ with a mean GY of 9.98 t ha^-1^ under FUT and 9.39 to 12.7 t ha^-1^ with a mean GY of 11.5 t ha^-1^ under FT treatment (P <0.001, Table 1).

The top three high-yielding genotypes under the FUT and FT treatments that were identified by the AMMI 1 analysis were G16, G19, and G15 and G23, G15 and G18, respectively (S1C and S1D Fig). Genotypes that showed combined better performances were G19, G5 and G24 and G19, G16 and G14 under the FUT and FT treatments, respectively (S2 Table). Locations L5, L4 and L3 performed better than the overall mean under FUT, whilst the performance of L1 and L2 was below the overall mean. In the case of the FT treatment, L5 and L4 performed better, whereas the performance of L1, L2 and L3 was below the overall mean (S1C and S1D Fig).

**Fig 6:**
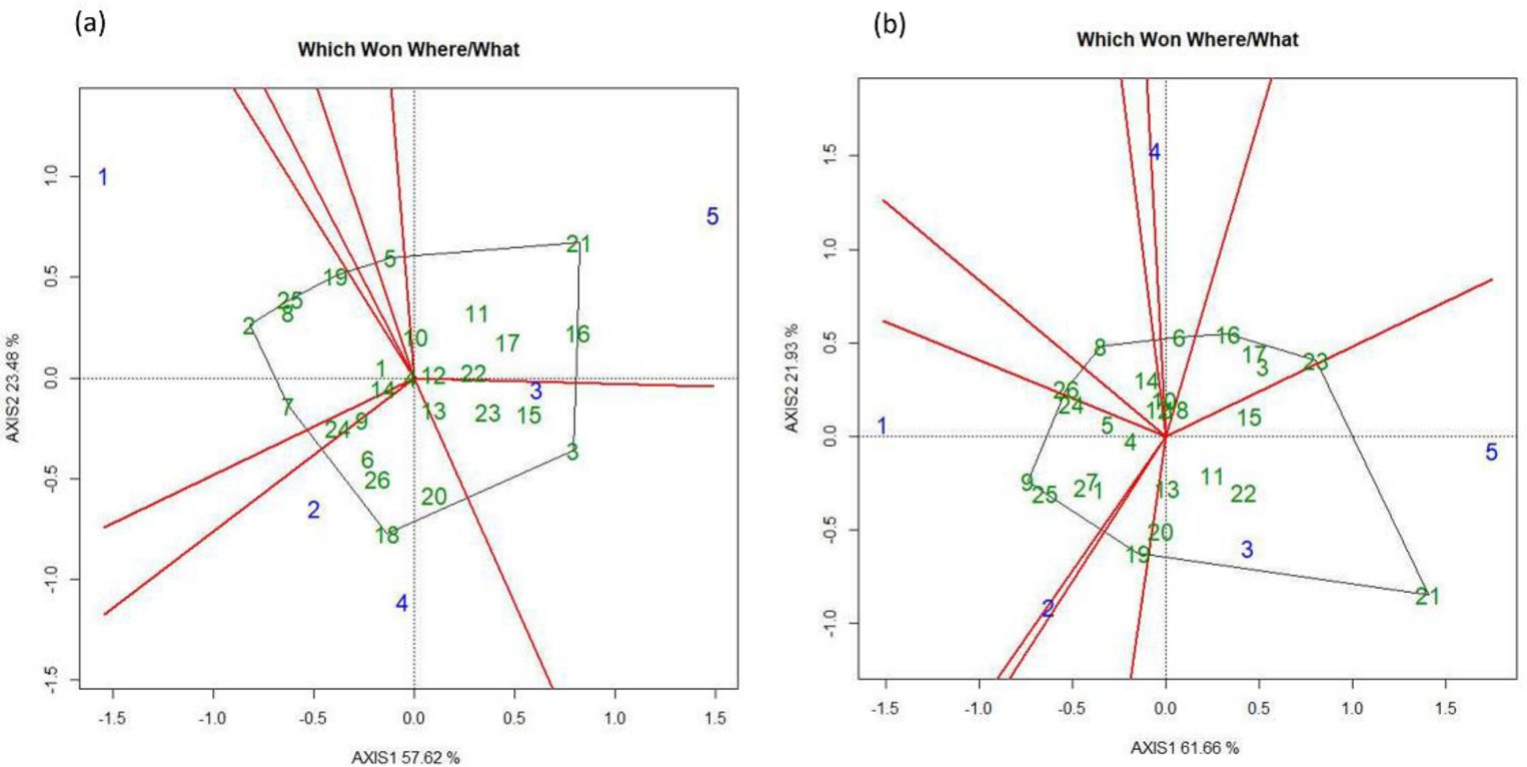
Showing GGE bi-plots for five locations for (a) fungicide untreated (FUT) and (b) treated (FT) treatments for wheat grain yield in 2017.

According to the GGE biplot for the FUT wheat, genotypes and locations were divided into eight major groups on the basis of their interactions and the best environment for achieving high GY. L1 and L5 were mainly two diverse environments, whilst L2 and L4were relatively similar. The genotype that group at the center of the plot represents those that exhibited the most stable performance at all the locations: G4 (WPB Oryx), G10 (WPB Scotch) and G12 (Skandus) (Fig 6A). When considering the FT treatment, the genotypes and locations were divided into nine groups. Locations L1, L2, and L4 were comparatively more diverse than each other whereas L3 and L5 were similar. The genotypes G12 (Skandus), G10 (WPB Scotch) and G18 (KW 440-2-14) (Fig 6B) were the most stable across locations, as shown by their grouping at the center of the plot.

#### 3.4.3 G × E 2018

Similar as in 2016 and 2017, genotypes showed significant variation in 2018 (P<0.001, Table 1). Average GY variation between different genotypes and locations ranged from 5.06 to 7.87 t ha^-1^ with a mean of 6.90 t ha^-1^ under FUT, whereas under FT it was ranged from 5.26 to 8.08 t ha^-1^ with a mean of 6.95 t ha^-1^ (P <0.001, Table 1).

The AMMI 1 analysis results shows top three high yielding genotypes under FUT and FT treatments were G10, G16, and G12 and G16, G10 and G9, respectively. The genotypes that showed the combined best performance for GY, stability and resistance were G19, G8 and G26 and G14, G8 and G19 under the FUT and FT treatments, respectively (S2 Table). The locations that performed better than the overall mean under FUT were L5 and L4 whereas crop performance at L2 and L1 was below the overall mean. The locations that performed better than the overall mean under FT treatment were L5 and L4 whereas the crop performance at L1 and L2 was below the overall mean (S1E and S1F Fig).

**Fig 7:**
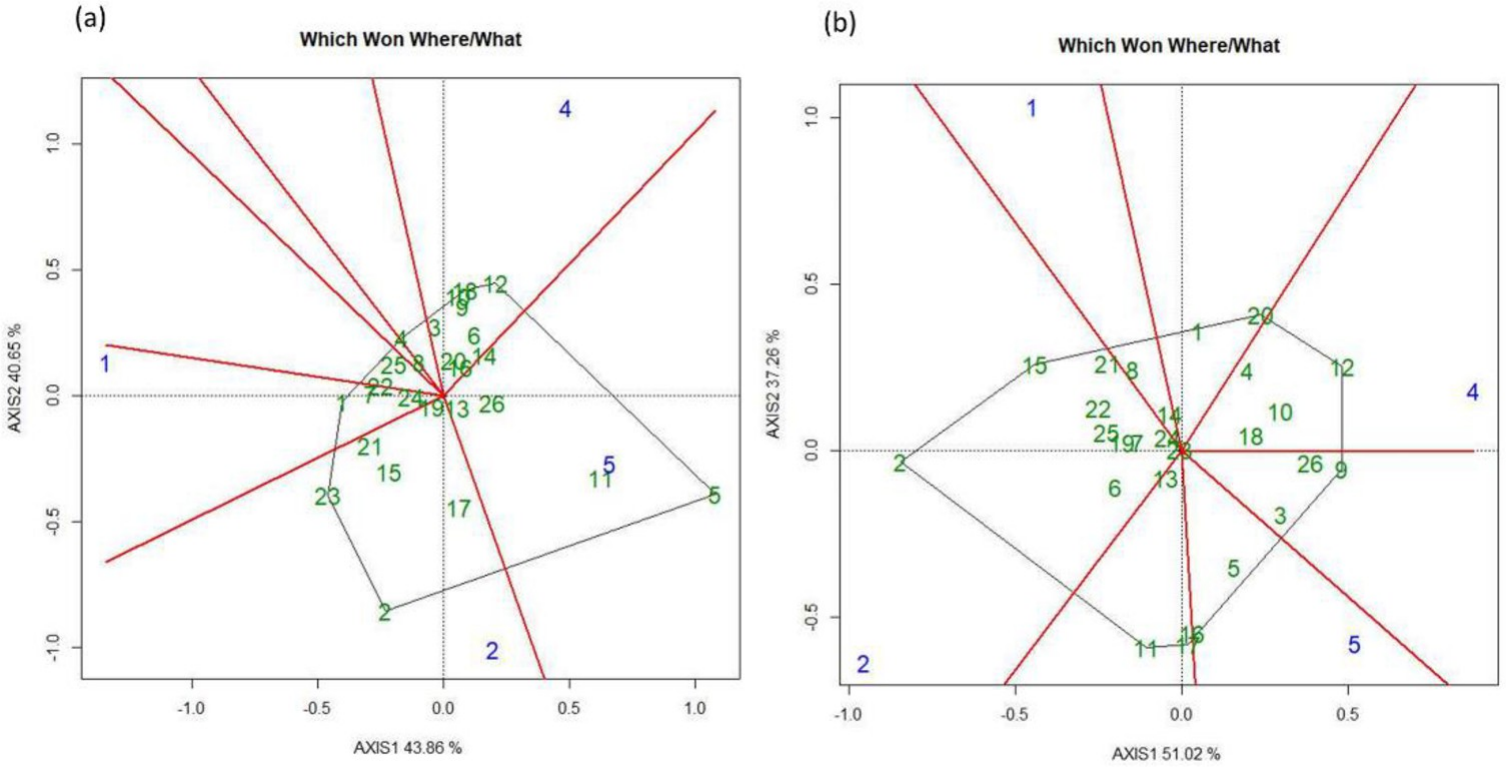
Showing GGE bi-plots for five locations for (a) fungicide untreated (FUT) and (b) treated (FT) treatments for wheat grain yield in 2018.

The GGE biplot for the FUT genotypes and locations were divided into seven groups. L1 and L2 were mainly diverse environments whereas L5 and L4 were relatively similar. The genotypes that showed stable performance at all the locations were G13 (SEC 526-07-2), G19 (Zenon) and G24 (SW 131324) (Fig 7A). In terms of dividing genotypes and locations into groups based on their performance and interaction with the local environment under FT, the genotypes were divided into nine groups. Locations L1 and L2, and L4 and L5 were grouped together and were comparatively similar. There was no location that was close to the center of the biplot. The genotypes grouped at the center and exhibited, overall, the most stable performance at all locations were G16 (KWS Spindrift), G13 (SEC 526-07-2) and G8 (WPB Scotch)(Fig 7B).

#### 3.4.4 G × E 2019

In 2019 there was significant phenotypic variation between the genotypes with respect to GY (P <0.001). Average GY across the genotypes and locations varied from 8.12 to 10.3 t ha^-1^ with a mean of 9.55 t ha^-1^ under FUT and 8.56 to 10.5 t ha^-1^ with a mean GY of 9.83 t ha^-1^ under FT treatment (P <0.001, Table 1).

The AMMI 1 analysis showed that genotypes G20, G5, and G15 and G5, G18 and G12 were the top three high-yielding varieties under the FUT and FT treatments, respectively (S1G and S1H Fig). The genotypes that exhibited the combined best performance were G14, G19 and G15, and G5, G13 and G15 under the FUT and FT treatments, respectively (S2 Table). For both treatments, locations L5 and L4 performed better than the overall mean whereas the crop performance at L2 and L3 was below the overall mean (S1G and S1H Fig).

**Fig 8:**
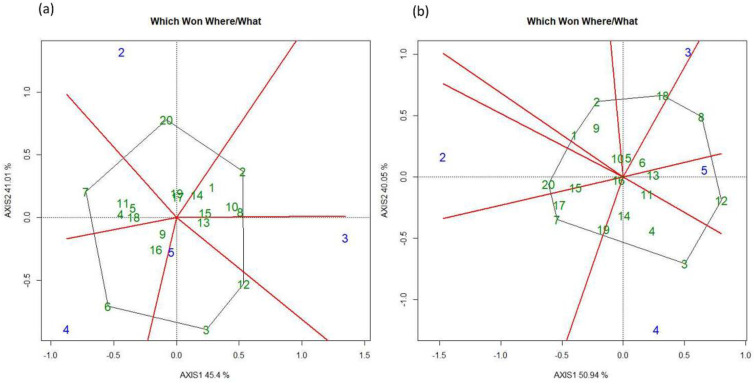
Showing GGE bi-plots for five locations for (a) fungicide untreated (FUT) and (b) treated (FT) treatments for wheat grain yield in 2019.

According to the GGE biplot, under FUT, genotypes and locations were divided into six groups. All the locations were relatively diverse than each other, and location L5 was close to the center of the biplot, indicating that most of the genotypes behave similarly with respect to GY at this location. There was no clear indication of genotypes that showed stable performance at all the locations (Fig 8A). For the FT treatment, genotypes and locations were divided into eight under this treatment, and were relatively diverse than each other. The genotypes that exhibited overall stable performance at all locations were G16 (SW 131323), G10 (Millie) and G5 (WPB Skye) (Fig 8B).

#### 3.4.5 G × E 2020

Overall in 2020, there was significant phenotypic variation between the genotypes with respect to GY (P <0.001, Table 1). The average GY across the genotypes and locations varied from 8.61 to 11.1 t ha^-1^ with a mean of 9.99 t ha^-1^ under FUT and 9.09 to 11.7 t ha^-1^ with a mean GY of 10.7 t ha^-1^ under FT treatment (Table 1; P <0.001). AMMI 1 analysis revealed G12, G11, and G13 under FUT and G11, G3 and G12 under FT as the top three high-yielding genotypes. Genotypes that showed the combined best performance for key selection traits GY, stability and resistance were G12, G12 and G6 and G12, G16 and G13 under the FUT and FT treatments, respectively (S2 Table). The locations that performed better than the overall mean under FUT were L3 and L5, whereas crop performance at L1 and L2 was below the overall mean. On the other hand, the locations that performed better than the overall mean under FT treatment were L5 and L3, whereas crop performance at L1 and L2 was below the overall mean (S1I and S1J Fig).

**Fig 9:**
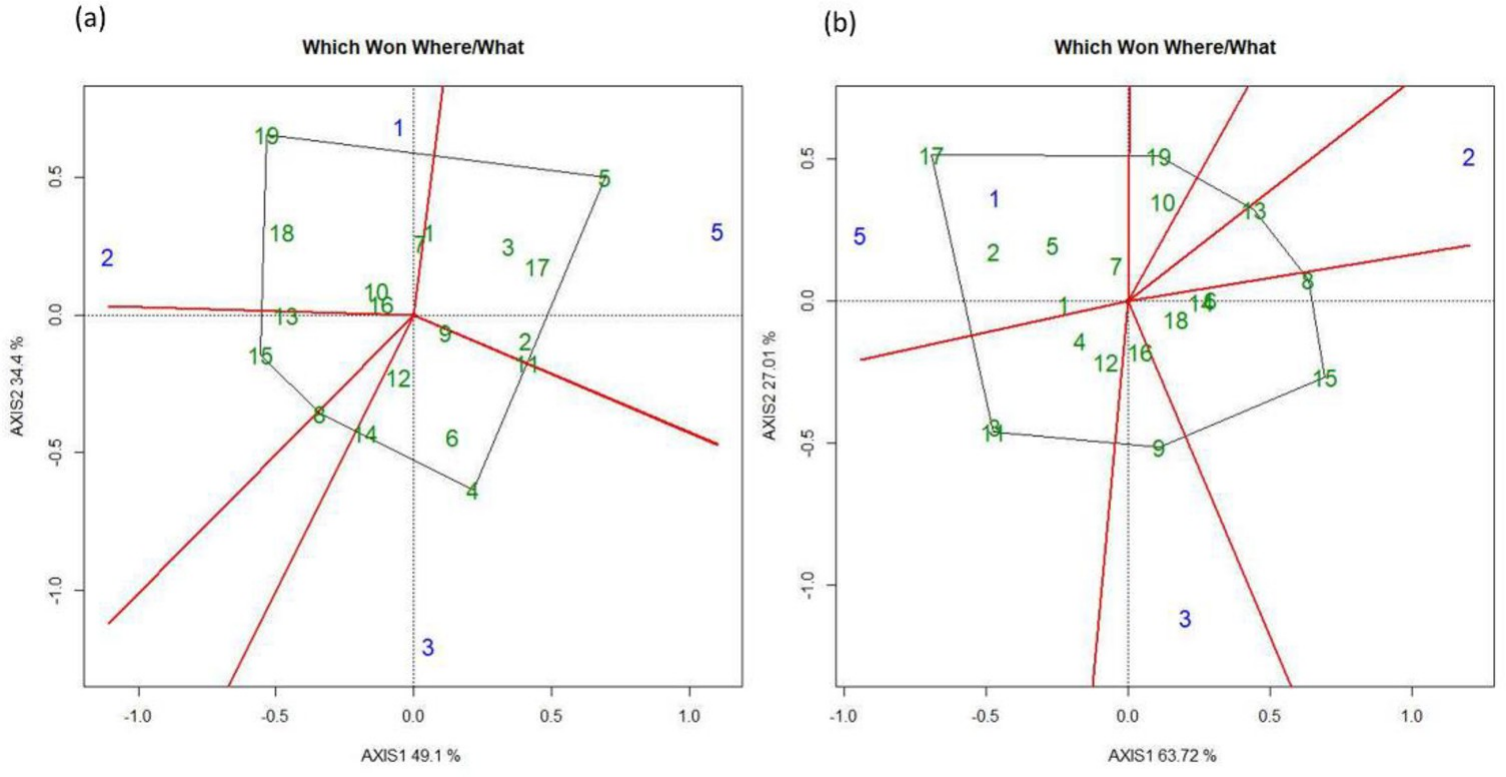
Showing GGE bi-plots for five locations for (a) fungicide untreated (FUT) and (b) treated (FT) treatments for wheat grain yield in 2020.

The GGE biplot analysis for the FUT treatment divided genotypes and locations based on their interaction into major five groups with respect to GY. Most of the locations were in divert groups and none was close to the center of the biplot. There were not many genotypes grouped at the center of the plot and only G16 and G10 showed stable performance at all locations (Fig 9A). For the FT treatment, the genotypes and locations were divided into seven groups. Locations L1 and L5 were comparatively close to each other, whereas L3 and L2 were more diverse than others. There were no clear genotypes grouped at the center of the plot (Fig 9B).

### 3.5 AMMI ANOVA of disease tolerance

The AMMI ANOVA for disease tolerance is shown in Table 2. AMMI analysis showed that there was a significant difference between treatments in 2016, 2017, and 2018 in relation to disease tolerance. Overall, in 2017, there was higher fungal infection compared to the other years, creating ideal conditions for the screening of disease tolerant genotypes. However, in the years 2018 and 2019, the relative difference in yield reduction under FUT than FT was very small. Averaging across the genotypes, the reduction in GY under FUT compared to FT was 0.88 t ha^-1^ (-9.41%) in 2016, 1.53 t ha^-1^ (-13.3%) in 2017 and 0.05 t ha^-1^ (-0.68%) in 2018.

AMMI analysis showed that there was a significant difference between the genotypes in 2016 and 2017. On average, proportional GY loss was lower for G4, G14 and G23 than for G17, G21 and G2 in 2016, indicating tolerant genotypes. Similarly, G24, G25 and G19 exhibited less yield reduction under FUT as of FT than for G21, G4 and G23 in 2017, indicating tolerance of fungal diseases.

There was a significant difference between all locations. Overall, in terms of main effects, the treatment effect was stronger in 2017 as compared to the other years and the location effect was stronger in 2016 and 2018. Considering IPCA, only IPCA 1 was significant across all years and IPCA 2 and the residual were not significant. IPCA 1 also explained more variation in disease tolerance in all years.

## 4. Discussion

Sweden, along with the other Nordic countries of Finland, Denmark, Iceland and Norway, is the most northern part of the globe in which field crops are grown. An increase in temperature due to climate change will have a greater adverse impact on wheat production in this part of the world because of the introduction of new plant diseases [10]. Developing new adaptable and resistant varieties is one way forward. The study of the interaction between genotype and location is, therefore, very important to allow breeders to develop the best strategies. In this study, our objective was to understand the effect of the environment along with fungal diseases on spring wheat production. We identified genotypes that exhibit tolerance to fungal diseases and are adaptable to specific or varied environments. We also identified locations that are optimal for spring wheat production in Sweden. In this study, we have used the same set of genotypes across all locations in each year and a different set of genotypes with four common genotypes across the years. The years included in this study exhibited a range of environmental variation, providing ideal conditions to study the genotype/environment interaction and its effects. For example, the years 2017, 2020 and 2016 were relatively normal in terms of yield production, with no drought stress, whilst 2018 had severe and drought and 2019 mild drought. This allowed us to compare different environmental scenarios in natural field conditions. Similarly, fungal infection could be examined in relation to different genotypes in natural field conditions. This study was also able to help in assessing the current strategy used by the Swedish national varietal trial evaluation for variety selection.

### 4.1 Grain yield and effect of environment on agricultural production regions of Sweden

Overall, the southern region of Sweden is traditionally seen as suitable for agricultural production. Here, the field trails was subdivided into four production regions (PR), two (PR1 and PR2) in the South zone and two (PR3 and PR4) in North zone. Overall across our five study years, the South zone was expected to produce a higher yield than North zone [19]; however, we did not see a big yield difference. One of the reasons could be because, during these years, the South zone was affected by droughts and heat stress. In particular, the heat stress and drought in 2018 reduced the yield significantly more in the South zone than in the North zone [20]. In addition, in 2018, the overall temperature was higher and this led to a shorter growing period and earlier physiological maturity [6]. This is a very common observation and mainly related to the physiological phenomenon where, due to higher temperatures and greater light intensity, plants increase their photosynthesis and respiration activities, thus reaching physiological maturity earlier. In addition, when plants sense stress, they tend to shorten their life cycle, reaching the reproductive stage earlier in order to produce seeds and thus continue the species’ survival. This is also an escape mechanism for plants from stress conditions like drought [21] and nutrient limitation [22]. Shortening the cropping period and increasing physiological activities are therefore not necessarily associated with an increase in yield, thus resulting in there being no great yield difference between the South and North zones. However, PR2 (part of the South zone) had the highest grain yield across all locations and years, and this could be explained by the fact that this region has ideal conditions for spring wheat growing in terms of temperate and rainfall.

Comparing the effect of fungicide treatment, we observed fluctuation in percent yield reduction due to fungicide treatment was observed depending on the year and disease severity. Also, previous studies from Sweden, report fluctuation in yield due to fungal disease for historic data from 432 spring wheat trials conducted in farmers’ fields between 1977 and 2005 [23]. Overall, 2017 had higher yield along with higher relative yield reduction in the no fungicide treatment compared to the FT treatment (13.3%). The results were PR2, where the overall yield was higher across all locations along with higher relative yield reduction when fungicide was not applied. These results could be just because of relative differences, or this may be associated with the fact that favorable conditions for crop growth also favor diseases [23]. We also noticed that across the locations and years, there was a 6.33% yield reduction with the highest reduction in 2017 (13.3%) and the lowest in 2018 (0.98%) in the FUT treatment (Table 1). As 2018 was affected by severe drought, overall disease pressure was low and there was not much yield difference between the two treatments. For diseases to thrive at higher temperatures, relative humidity is also important. In Sweden, crop production is regularly affected by early and late frost but generally the area suffers from fewer plant diseases mainly because of its cold weather, which is unfavorable for pathogens and pests [24]. However, an increasing temperature in the future will change this scenario and the environment will be more favorable for diseases, as shown by simulations of future agricultural conditions [14]. To address this situation, more studies will be required to elucidate the G x E interaction with respect to plant disease, along with phenotyping and evaluating more crop germplasms, and developing appropriate management systems.

In this study, we found that there was a strong negative correlation between GY and temperature, showing that higher temperature has an adverse effect on yield. This can be understood in relation to the positive correlation between crop duration and GY, where genotypes that have a longer growing period produce a greater yield than early maturing genotypes. This relationship can be explained by the physiological fact that a longer growth period give more time for photosynthesis and, thus, more assimilation, resulting increased amount of starch accumulation in the grain and thereby higher grain yield [22]. In the future, the temperature in Scandinavian counties will increase due to climate change. In this case, a quick solution to this problem will be to introduce varieties from Southern European counties like France and Spain that have more stable GY under relatively higher temperatures. Moreover, one of the key observations of our current experiment was that GY is more strongly influenced by temperature than rainfall/drought. This shows that in the near future, spring wheat yield will be more negatively affected by heat stress than drought stress. The most probable explanation is that drought can be escaped by plants through early maturity, however, heat stress at critical growth stages like flowering will affect grain yield significantly. In this study, significant yield reduction above 17°C was observed across the locations (Fig 1B) suggesting that to achieve sustainable yield in the future, breeders have to use the benchmark of heat tolerance varieties of above 17°C in Nordic conditions. Moreover, with increasing temperatures, we have seen lesser disease severity. However, in the future pathogen also expected to evolve, and therefore, breeders will need to develop varieties that are heat tolerant and resistant to diseases. Semenov and Shewry [25] studied the effect of heat and drought on European winter wheat using a simulation model, Sirius17, while considering various climate change scenarios. This model also predicted that heat stress will be more important than drought in limiting the grain yield across Europe. Therefore, the breeding strategy for Swedish spring wheat could be focused on developing heat-tolerant genotypes rather than drought tolerance. When developing such genotypes, relevant physiological traits should be targeted in breeding programs. For example, Cossani and Reynolds [26] proposed the traits base model that will be valuable for heat tolerance breeding. In this model, when water and nutrients are not limiting factors, then the yield is the function of light interception, radiation use efficiency and partitioning of total assimilation. Therefore, to improve light interception and radiation use efficiency, target traits could be rapid ground cover, stay-green, spike photosynthesis, respiration, membrane thermostability, and rubisco efficiency; in contrast, to improve water use efficiency, traits like water uptake and transpiration regulation will be important.

### 4.2 Genotype × Environment interactions

As the genotypes selected for individual years were the same across all five locations, any difference in response of genotype with respect to yield was mainly because of the G x E interaction. The ANOVA for the split-plot experimental design shows that in most of the years there were significant differences with respect to genotype, location and their interactions. Considering the overall effects across all years, yield is strongly influenced by the environment of the location. This was especially so in 2018 and 2019, when the environmental effect, mainly related to severe drought, was very strong and therefore, the fungicide treatment effect was less apparent. It is always very challenging for a breeder to make a selection when there is a strong effect of the environment. In the experiments considered herein, there were four or five locations examined in each year, thus helping in selecting stable genotypes that showed consistently higher GY. The fungicide treatment effect was relatively stronger in the years 2016, 2017 and 2020, and therefore, selecting genotypes that are resistant to fungal diseases was focused on only in these years.

Overall, the genotypes that performed better for GY under the FT treatment also performed better under the FUT treatment in all years, across the locations, indicating that over the breeding period high yielding genotypes were also selected for disease resistance. In terms of individual genotypes, G4 (KWS Alderon), G14 (WPB 09SW025-11) and G23 (SW 11360) in 2016, G24 (SW 11360), G25 (Millie) and G19 (SEC 526-07-2) in 2017, and G19 (WPB 13SW976-01), G12 (Levels) and G18 (SW 141011) in 2020 were the most disease-resistant and could be used further in breeding new resistant cultivars. These varieties were developed by breeding companies as well as the Swedish national breeding institute and are popular in Scandinavian countries. Among the genotypes that showed higher yields across the years, the most common come from KWS, including KWS Alderon and KWS Cochise, showing their importance in the Scandinavian environment. In this study, we were also looking for genotypes that showed combined better performance for disease resistance and had stable and higher yields across the locations. Using the ranking of these three selection criteria, we give a combined ranking and use it as a selection index. With this combined selection index, we suggest that genotypes KWS Alderon, SEC 526-07-2, Thorus, WPB Skye, and WPB 13SW976-01 are the best for stable yield across Sweden when combined with fungicide application. At present, when people are more health and environmentally conscious, there is a growing concern about fungicide application in agricultural food production. Thus, farmers are looking for varieties that need minimum fungicide application. For example, wheat growers who farm according to organic principles or focus on sustainable agriculture production, should consider the genotypes WPB Avonmore, SEC 526-07-2, Zenon, and SW 131323, which deliver a better yield without the use of fungicides.

In the present investigation, experiments were conducted as part of the program of Swedish national trials, in which breeders test a set of advanced breeding lines in different locations representing Swedish agricultural growing regions. However, there are only limited genotypes that are grown repeatedly at the same location for multiple years. To evaluate the better and more stable genotypes at a particular location or within a zone, it would be valuable to crop the same set of genotypes in each location or zone for two or three years to determine the genotype stability for yield. As the crop growth period is different in the South and North agricultural growing zones, the breeding program should focus on developing varieties separately for each zone. The importance of breeding varieties according to zone in Swedish national trials was also highlighted for winter wheat and spring barley by Buntaran, Piepho [27]. In contrast, Yan and Rajcan [28] have suggested that in soybean trials single-year data could be sufficient to identify the best and worst genotypes in Canada. In the current breeding program, the focus is more on yield traits but there is potential to include physiological traits that can help in better understanding the mechanism responsible for higher yield under different climatic scenarios. This could help in developing ideotype and simultaneous trait selection in pre-breeding and breeding.

## 5. Conclusions and future perspectives

As a consequence of climate change, the temperature and rainfall in the South and North zones of the Swedish agricultural production regions will change. This data clearly shows that in the near future, heat stress will be the main yield-limiting factor in spring wheat production. Moreover, frequent droughts will add more limitations to spring wheat yield production as the traditional varieties are adapted to high soil moisture conditions in the Nordic region. When the conditions favor higher yields, fungal diseases are also positively affected, thus having an adverse impact on yield if the crop is not sprayed with fungicides. There is a very clear need to develop a breeding strategy including the simultaneous selection of traits that are responsible for minimizing heat stress and increasing disease resistance. Understanding how genotypes interact within both the micro-environment like South-West or South-East but also the mega-environment like South Zone or North Zone is essential. As the crop growing period and timing and duration of drought or heat stress will change according to the crop growing zone, the selection of stress tolerant traits should focus on early, mid and late droughts in pre-breeding programs. In terms of identifying disease resistant genotypes, resistance screening will demand new phenotyping techniques, in which thousands of breeding lines can be screened at different locations under field conditions. Genotype screening for disease resistance could be conducted simultaneously under artificial conditions if appropriate natural conditions are not available.

## Supporting information

S1 Table Showing year, location code and name, zone, regions, previous crop, sowing and harvesting data, crop duration, mean temperature, thermal time (TT) and rainfall, grain yield under fungicide untreated (FUT) and treated (FT) treatment, and percent yield reduction across all locations for five years. (DOCX)

S2 Table Genotype name (Geno), genotype ID (G ID), ranking for higher grain yield (GY Rk), ranking for AMMI stability values (StRk) under fungicide untreated (FUT) and treated (FT) treatment and ranking for percent GY reduction under FUT (% GY red rank). (DOCX)

S1 Fig Showing AMMI-1 biplot for fungicide untreated (FUT) and fungicide treated (FT) treatment for wheat grain yield for all years. (a) 2016 FUT, (b) 2016 FT, (c) 2017 FUT, (d) 2017 FT, (e) 2018 FUT, (f) 2018 FT, (g) 2019 FUT, (h) 2019 FT, (i) 2020 FUT, (j) 2020 FT.
(DOCX)
