# Supplementary material for: Genotype and environment interaction study shows fungal diseases and heat stress are detrimental to spring wheat production in Sweden
Source: PLoS One. 2023 May 10;18(5):e0285565. doi: 10.1371/journal.pone.0285565 (PMC10171613; doi:10.1371/journal.pone.0285565)
Supplement: S1 Table — (DOCX) [file pone.0285565.s001.docx]

**S Table 1. Showing year, location code and name, zone, regions, previous crop, sowing and harvesting data, crop duration, mean temperature, thermal time (TT) and rainfall, grain yield (GY) under fungicide untreated (FUT) and treated (FT) treatment, and percent yield reduction (%GY red) across all locations for five years.**

| **Year** | **Code** | **Location Name** | **Zone** | **Regions** | **Previous Crop** | **Sowing date** | **Harvesting date** | **Crop Duration** | **Mean Temp °C** | **TT °CD** | **Total Rainfall mm** | **GY t ha^-1^**  **FUT** | **GY t ha^-1^**  **FT** | **%GY Red** |
| --- | --- | --- | --- | --- | --- | --- | --- | --- | --- | --- | --- | --- | --- | --- |
| 2016 | L1 | Eskilstorpsgård | South | PR2 | Sugar beet | 04/04/2016 | 26/08/2016 | 144 | 14.8 | 2142 | 231.2 | 9.42 | 10.68 | 11.75 |
|  | L2 | Stora Uppåkravägen | South | PR1 | Sugar beet | 02/04/2016 | 24/08/2016 | 144 | 13.8 | 1997 | 291.6 | 8.06 | 8.22 | 1.98 |
|  | L3 | Giresta, Staby Säteri | North | PR4 | Spring barley | 06/04/2016 | 16/09/2016 | 163 | 14.1 | 2316 | 280.6 | 8.96 | 9.77 | 8.27 |
|  | L4 | Nybble | North | PR4 | Spring barley | 05/04/2016 | 06/09/2016 | 154 | 13.7 | 2127 | 313.8 | 9.89 | 11.51 | 14.07 |
|  | L5 | Klostergården | North | PR3 | Potato | 15/04/2016 | 18/08/2016 | 125 | 13.9 | 1753 | 205.2 | 6.46 | 7.04 | 8.25 |
|  |  |  |  |  |  |  | **Mean** | **146** | **14.1** | **2067** | **264.5** | **8.56** | **9.44** | **8.86** |
|  |  |  |  |  |  |  |  |  |  |  |  |  |  |  |
| 2017 | L1 | Eskilstorpsgård | South | PR2 | Sugar beet | 02/04/2017 | 26/08/2017 | 146 | 13.3 | 1958 | 278.6 | 7.52 | 10.51 | 28.39 |
|  | L2 | Stora Uppåkravägen | South | PR1 | Sugar beet | 23/04/2017 | 03/09/2017 | 133 | 15.2 | 2039 | 245.8 | 8.84 | 10.25 | 13.81 |
|  | L3 | Giresta, Staby Säteri | North | PR4 |  | 16/03/2017 | 07/09/2017 | 175 | 10.5 | 1947 | 173.2 | 10.42 | 11.18 | 6.78 |
|  | L4 | Nybble | North | PR4 |  | 16/03/2017 | 18/09/2017 | 186 | 12.0 | 2237 | 364.2 | 11.02 | 12.29 | 10.32 |
|  | L5 | Klostergården | North | PR3 | Winter wheat | 04/05/2017 | 18/09/2017 | 118 | 15.0 | 1780 | 205.1 | 12.11 | 13.34 | 9.19 |
|  |  |  |  |  |  |  | **Mean** | **155** | **13.2** | **1992** | **253.4** | **9.98** | **11.5** | **13.7** |
|  |  |  |  |  |  |  |  |  |  |  |  |  |  |  |
| 2018 | L2 | Multorp, Sal | North | PR3 | Winter wheat | 06/05/2018 | 26/08/2018 | 112 | 17.8 | 2014 | 174.7 | 5.71 | 5.49 | -3.98 |
|  | L1 | Stora Uppåkravägen | South | PR1 | Sugar beet | 22/04/2018 | 15/08/2018 | 115 | 17.5 | 2411 | 143.0 | 4.59 | 4.50 | -1.84 |
|  | L4 | Nybble | North | PR4 | Spring barley | 30/04/2018 | 07/08/2018 | 99 | 18.0 | 1797 | 110.8 | 7.31 | 7.46 | 2.03 |
|  | L5 | Klostergården | North | PR3 | Winter wheat | 18/04/2018 | 03/08/2018 | 107 | 16.9 | 1820 | 87.8 | 10.00 | 10.34 | 3.28 |
|  |  |  |  |  |  |  | **Mean** | **108** | **17.5** | **2010** | **129.1** | **6.90** | **6.95** | **-0.1** |
|  |  |  |  |  |  |  |  |  |  |  |  |  |  |  |
| 2019 | L2 | Kampetorp | North | PR3 | Spring barley | 17/04/2019 | 15/09/2019 | 151 | 14.5 | 2209 | 363.0 | 9.07 | 9.35 | 3.00 |
|  | L1 | Stora Uppåkravägen | South | PR1 | Sugar beet | 12/04/2019 | 15/09/2019 | 156 | 14.5 | 1813 | 163.6 |  |  |  |
|  | L3 | Giresta, Staby Säteri | North | PR4 | Seed flax | 30/04/2019 | 17/09/2019 | 140 | 14.6 | 2063 | 315.6 | 8.20 | 8.21 | 0.15 |
|  | L5 | Nybble | North | PR4 | Spring barley | 08/04/2019 | 09/09/2019 | 154 | 14.3 | 2209 | 308.4 | 10.70 | 11.39 | 6.09 |
|  | L4 | Klostergården | North | PR3 | Winter wheat | 09/04/2019 | 29/08/2019 | 142 | 13.8 | 1974 | 259.0 | 10.25 | 10.36 | 1.05 |
|  |  |  |  |  |  |  | **Mean** | **149** | **14.3** | **2054** | **281.9** | **9.55** | **9.83** | **2.57** |
|  |  |  |  |  |  |  |  |  |  |  |  |  |  |  |
| 2020 | L2 | Flo, Bragnum | North | PR3 | Spring wheat | 21/04/2020 | 11/09/2020 | 143 | 14.5 | 2089 | 296.0 | 7.89 | 8.42 | 6.26 |
|  | L1 | Nyhem | South | PR1 | Sugar beet | 30/03/2020 | 17/08/2020 | 140 | 14.0 | 1971 | 195.2 | 9.46 | 10.34 | 8.54 |
|  | L3 | Giresta, Staby Säteri | North | PR4 | Spring barley | 28/04/2020 | 23/09/2020 | 148 | 14.3 | 2126 | 207.6 | 10.49 | 11.43 | 8.27 |
|  | L4 | Klostergården | North | PR3 | Winter wheat | 17/04/2020 | 24/08/2020 | 129 | 13.7 | 1775 | 226.4 | 12.12 | 12.50 | 3.09 |
|  |  |  |  |  |  |  | **Mean** | **140** | **14.1** | **1990** | **231.1** | **10.0** | **10.7** | **6.54** |
|  |  |  |  |  |  |  |  |  |  |  |  |  |  |  |
|  | South |  |  |  |  |  | **Mean** | **137** | **14.8** | **2086** | **230.9** | **7.98** | **9.08** | **10.4** |
|  | North |  |  |  |  |  | **Mean** | **142** | **14.5** | **2015** | **243.0** | **9.41** | **9.69** | **5.38** |
