# Supplementary material for: Genotype and environment interaction study shows fungal diseases and heat stress are detrimental to spring wheat production in Sweden
Source: PLoS One. 2023 May 10;18(5):e0285565. doi: 10.1371/journal.pone.0285565 (PMC10171613; doi:10.1371/journal.pone.0285565)
Supplement: S2 Table — (DOCX) [file pone.0285565.s002.docx]

**S Table 2. Genotype name (Geno), genotype ID (G ID), ranking for higher grain yield (GY Rk), ranking for AMMI stability values (StRk) under fungicide untreated (FUT) and treated (FT) treatment and ranking for percent GY reduction under FUT (% GY Red Rank).**

| **ID** | **Genotype Name** | **Grain Yield Rank** | | **% GY**  **red Rank** | **AMMI Stability Rank** | | **Combine Rank** | |
| --- | --- | --- | --- | --- | --- | --- | --- | --- |
|  |  | **FUT** | **FT** |  | **FUT** | **FT** | **FUT** | **FT** |
|  | **2016** |  |  |  |  |  |  |  |
| G4 | KWS Alderon | 1 | 3 | 1 | 25 | 13 | 4 | 1 |
| G14 | WPB 09SW025-11 | 2 | 5 | 2 | 18 | 21 | 3 | 4 |
| G25 | WPB 09SW025 | 3 | 1 | 11 | 19 | 20 | 9 | 8 |
| G13 | KWS Cochise | 4 | 2 | 20 | 17 | 24 | 16 | 18 |
| G8 | WPB Skye | 5 | 7 | 14 | 1 | 16 | 2 | 11 |
| G12 | KWS 302 | 6 | 8 | 16 | 8 | 23 | 7 | 19 |
| G19 | WPB Avonmore | 7 | 13 | 4 | 7 | 3 | 1 | 2 |
| G20 | Skandus | 8 | 4 | 21 | 24 | 1 | 22 | 3 |
| G6 | WPB Oryx | 9 | 6 | 22 | 3 | 9 | 10 | 11 |
| G9 | Miramis | 10 | 12 | 9 | 10 | 14 | 5 | 10 |
| G15 | Bumble | 11 | 16 | 6 | 20 | 18 | 14 | 15 |
| G7 | Happy | 12 | 14 | 8 | 11 | 11 | 8 | 9 |
| G18 | WPB Scotch | 13 | 9 | 19 | 2 | 15 | 10 | 16 |
| G10 | Rohan | 14 | 11 | 15 | 5 | 4 | 10 | 6 |
| G5 | Countess | 15 | 17 | 7 | 15 | 6 | 14 | 6 |
| G11 | KWS Healy | 16 | 10 | 18 | 16 | 10 | 19 | 13 |
| G23 | SW 11360 | 17 | 20 | 3 | 9 | 5 | 5 | 4 |
| G16 | SW 01278 | 18 | 18 | 13 | 4 | 12 | 13 | 16 |
| G1 | Diskett SW 45456 | 19 | 19 | 17 | 12 | 2 | 18 | 13 |
| G21 | STRU 093735s5 | 20 | 15 | 24 | 14 | 8 | 24 | 19 |
| G24 | SW 11230 | 21 | 21 | 10 | 13 | 22 | 17 | 23 |
| G22 | CH211.13701 | 22 | 23 | 12 | 22 | 25 | 23 | 24 |
| G2 | Triso | 23 | 22 | 23 | 6 | 7 | 20 | 22 |
| G3 | Quarna | 24 | 25 | 5 | 23 | 17 | 20 | 19 |
| G17 | Kreivi | 25 | 24 | 25 | 21 | 19 | 25 | 25 |
|  |  |  |  |  |  |  |  |  |
|  | **2017** |  |  |  |  |  |  |  |
| G16 | WPB 09SW025-11 | 1 | 4 | 8 | 24 | 14 | 7 | 2 |
| G19 | SEC 526-07-2 | 2 | 8 | 3 | 17 | 10 | 1 | 1 |
| G15 | KWS Cochise | 3 | 2 | 17 | 19 | 17 | 12 | 10 |
| G23 | WPB 09SW025-09 | 4 | 1 | 24 | 15 | 25 | 15 | 22 |
| G3 | KWS Alderon | 5 | 9 | 9 | 23 | 21 | 9 | 13 |
| G17 | Amantis | 6 | 10 | 10 | 18 | 19 | 8 | 13 |
| G18 | KW 440-2-14 | 7 | 3 | 23 | 14 | 3 | 17 | 5 |
| G5 | Happy | 8 | 13 | 7 | 8 | 11 | 2 | 7 |
| G24 | SW 11360 | 9 | 21 | 1 | 16 | 20 | 3 | 19 |
| G14 | KWS 302 | 10 | 6 | 16 | 5 | 5 | 6 | 3 |
| G8 | Bumble | 11 | 17 | 6 | 22 | 13 | 12 | 10 |
| G12 | Skandus | 12 | 5 | 22 | 3 | 1 | 9 | 4 |
| G11 | WPB Avonmore | 13 | 14 | 12 | 13 | 9 | 11 | 9 |
| G22 | Sibelius | 14 | 12 | 13 | 12 | 16 | 12 | 17 |
| G10 | WPB Scotch | 15 | 16 | 11 | 2 | 2 | 4 | 5 |
| G6 | WPB Skye | 16 | 11 | 20 | 11 | 8 | 23 | 13 |
| G20 | Quintus | 17 | 20 | 5 | 7 | 6 | 5 | 7 |
| G4 | WPB Oryx | 18 | 7 | 25 | 1 | 7 | 17 | 13 |
| G7 | Rohan | 19 | 22 | 4 | 20 | 15 | 15 | 17 |
| G26 | Flippen | 20 | 18 | 15 | 10 | 22 | 21 | 23 |
| G13 | KWS Healy | 21 | 15 | 19 | 4 | 4 | 17 | 12 |
| G25 | Millie | 22 | 24 | 2 | 21 | 23 | 21 | 20 |
| G9 | SW 01278 | 23 | 19 | 21 | 9 | 24 | 24 | 25 |
| G1 | Diskett SW 45456 | 24 | 23 | 14 | 6 | 12 | 17 | 20 |
| G2 | Quarna | 25 | 26 | 18 | 25 | 18 | 25 | 24 |
| G21 | Duramonte | 26 | 25 | 26 | 26 | 26 | 26 | 26 |
|  |  |  |  |  |  |  |  |  |
|  | **2018** |  |  |  |  |  |  |  |
| G10 | KWS Cochise | 1 | 2 | 14 | 18 | 16 | 11 | 5 |
| G12 | KW 440-2-14 | 2 | 5 | 8 | 22 | 25 | 6 | 14 |
| G26 | WPB 11SW250-10 | 3 | 6 | 11 | 7 | 19 | 2 | 11 |
| G16 | KWS Spindrift | 4 | 1 | 25 | 3 | 20 | 6 | 18 |
| G9 | Skandus | 5 | 3 | 24 | 15 | 24 | 17 | 23 |
| G3 | WPB Oryx | 6 | 4 | 19 | 12 | 17 | 14 | 15 |
| G17 | Thorus | 7 | 10 | 5 | 21 | 21 | 11 | 11 |
| G18 | KWS W353 | 8 | 8 | 18 | 19 | 7 | 19 | 8 |
| G8 | WPB Scotch | 9 | 11 | 6 | 6 | 10 | 2 | 2 |
| G11 | Amantis | 10 | 9 | 15 | 24 | 22 | 20 | 18 |
| G14 | Sibelius | 11 | 17 | 3 | 8 | 4 | 4 | 1 |
| G19 | Zenon | 12 | 19 | 4 | 1 | 6 | 1 | 3 |
| G22 | Millie (SW 21279) | 13 | 20 | 2 | 10 | 11 | 5 | 8 |
| G7 | SW 01278 | 14 | 15 | 12 | 14 | 5 | 15 | 5 |
| G24 | SW 131324 | 15 | 14 | 13 | 4 | 2 | 6 | 3 |
| G13 | SEC 526-07-2 | 16 | 12 | 17 | 2 | 3 | 13 | 5 |
| G20 | Flippen (SW 11361) | 17 | 18 | 10 | 5 | 18 | 6 | 18 |
| G5 | WPB Skye | 18 | 7 | 26 | 26 | 15 | 25 | 22 |
| G4 | Happy (SW 91003) | 19 | 13 | 21 | 13 | 12 | 21 | 18 |
| G23 | SW 131323 | 20 | 15 | 20 | 23 | 1 | 23 | 11 |
| G21 | SW 11360 | 21 | 21 | 7 | 16 | 14 | 17 | 17 |
| G25 | Roxette (SW 11088) | 22 | 24 | 1 | 9 | 9 | 6 | 10 |
| G6 | Bumble (SW 01121) | 23 | 23 | 9 | 11 | 8 | 16 | 15 |
| G1 | Diskett SW 45456 | 24 | 22 | 22 | 20 | 13 | 24 | 24 |
| G15 | Mirakel | 25 | 25 | 16 | 17 | 23 | 22 | 25 |
| G2 | Quarna | 26 | 26 | 23 | 25 | 26 | 26 | 26 |
|  |  |  |  |  |  |  |  |  |
|  | **2019** |  |  |  |  |  |  |  |
| G20 | SG S1483-16 | 1 | 8 | 2 | 16 | 16 | 4 | 8 |
| G5 | WPB Skye | 2 | 1 | 12 | 9 | 3 | 6 | 1 |
| G15 | KWS 555-3-15 | 3 | 4 | 9 | 6 | 10 | 3 | 3 |
| G14 | Zenon | 4 | 16 | 1 | 5 | 7 | 1 | 4 |
| G18 | WPB 11SW250-10 | 5 | 2 | 14 | 8 | 17 | 9 | 11 |
| G3 | WPB Oryx | 6 | 10 | 7 | 20 | 19 | 12 | 14 |
| G11 | Flippen | 7 | 13 | 6 | 11 | 5 | 7 | 4 |
| G19 | SW 21269 | 8 | 14 | 3 | 3 | 8 | 2 | 7 |
| G13 | Thorus | 9 | 6 | 8 | 4 | 6 | 5 | 2 |
| G12 | KWS Spindrift | 10 | 3 | 18 | 17 | 20 | 17 | 18 |
| G8 | Amantis | 11 | 5 | 11 | 14 | 18 | 13 | 13 |
| G7 | SW 01278 | 12 | 11 | 10 | 18 | 15 | 14 | 14 |
| G9 | Sibelius | 13 | 7 | 16 | 2 | 9 | 10 | 9 |
| G4 | Happy | 14 | 9 | 17 | 12 | 11 | 15 | 16 |
| G10 | Millie | 15 | 18 | 4 | 13 | 2 | 11 | 4 |
| G6 | Bumble | 16 | 15 | 13 | 19 | 4 | 18 | 9 |
| G16 | SW 131323 | 17 | 12 | 20 | 7 | 1 | 16 | 11 |
| G17 | Roxette | 18 | 19 | 5 | 1 | 14 | 7 | 17 |
| G1 | Diskett SW 45456 | 19 | 17 | 19 | 10 | 12 | 18 | 19 |
| G2 | Quarna | 20 | 20 | 15 | 15 | 13 | 20 | 19 |
|  |  |  |  |  |  |  |  |  |
|  | **2020** |  |  |  |  |  |  |  |
| G12 | Levels | 1 | 2 | 2 | 4 | 3 | 1 | 1 |
| G11 | KW 310-3-17 | 2 | 1 | 16 | 12 | 16 | 8 | 11 |
| G13 | KW 362-2-17 | 3 | 7 | 6 | 13 | 13 | 3 | 6 |
| G19 | WPB 13SW976-01 | 4 | 4 | 12 | 9 | 15 | 5 | 10 |
| G16 | KW 323-3-17 | 5 | 10 | 3 | 18 | 8 | 7 | 3 |
| G3 | WPB Skye | 6 | 3 | 19 | 7 | 12 | 12 | 12 |
| G9 | SW 131323 | 7 | 8 | 13 | 15 | 5 | 13 | 6 |
| G5 | Sibelius | 8 | 5 | 17 | 5 | 1 | 8 | 5 |
| G6 | Flippen | 9 | 11 | 8 | 1 | 2 | 2 | 3 |
| G7 | Thorus | 10 | 6 | 18 | 19 | 11 | 19 | 14 |
| G4 | Happy | 11 | 13 | 4 | 8 | 10 | 4 | 8 |
| G14 | SW 21269 | 12 | 9 | 11 | 2 | 9 | 5 | 9 |
| G18 | SW 141011 | 13 | 15 | 1 | 16 | 4 | 8 | 2 |
| G8 | Zenon | 14 | 12 | 15 | 11 | 17 | 17 | 19 |
| G15 | SW 141568 | 15 | 14 | 9 | 17 | 18 | 18 | 17 |
| G17 | KW 459-2-18 | 16 | 17 | 5 | 14 | 19 | 13 | 17 |
| G1 | Diskett SW 45456 | 17 | 16 | 14 | 6 | 7 | 16 | 15 |
| G10 | Roxette | 18 | 18 | 10 | 3 | 6 | 11 | 12 |
| G2 | Quarna | 19 | 19 | 7 | 10 | 14 | 15 | 16 |
