# Supplementary material for: Genotype and environment interaction study shows fungal diseases and heat stress are detrimental to spring wheat production in Sweden
Source: PLoS One. 2023 May 10;18(5):e0285565. doi: 10.1371/journal.pone.0285565 (PMC10171613; doi:10.1371/journal.pone.0285565)
Supplement: S1 Fig — (a) 2016 FUT, (b) 2016 FT, (c) 2017 FUT, (d) 2017 FT, (e) 2018 FUT, (f) 2018 FT, (g) 2019 FUT, (h) 2019 FT, (i) 2020 FUT, (j) 2020 FT. (DOCX) [file pone.0285565.s003.docx]

(a) (b) (c)


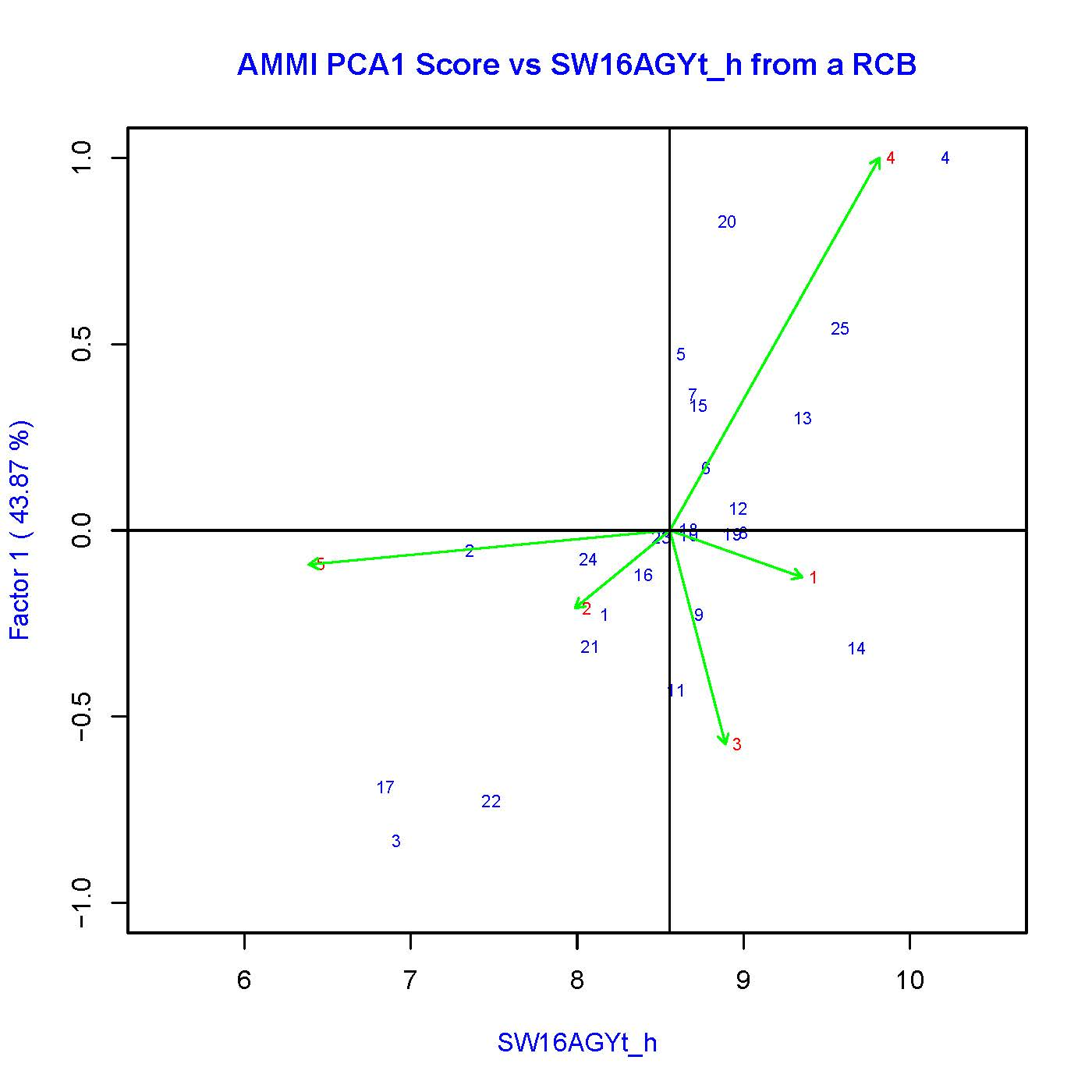

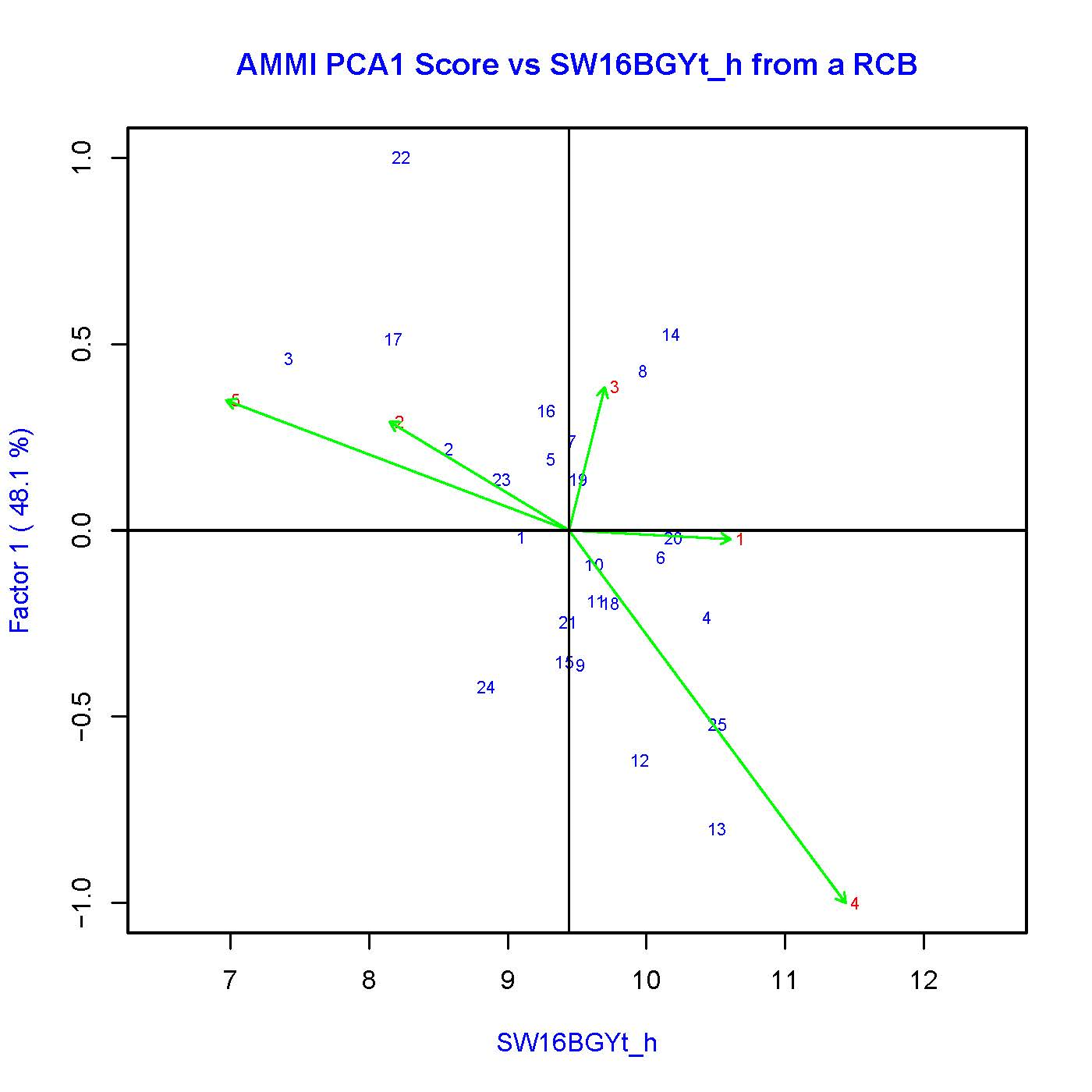

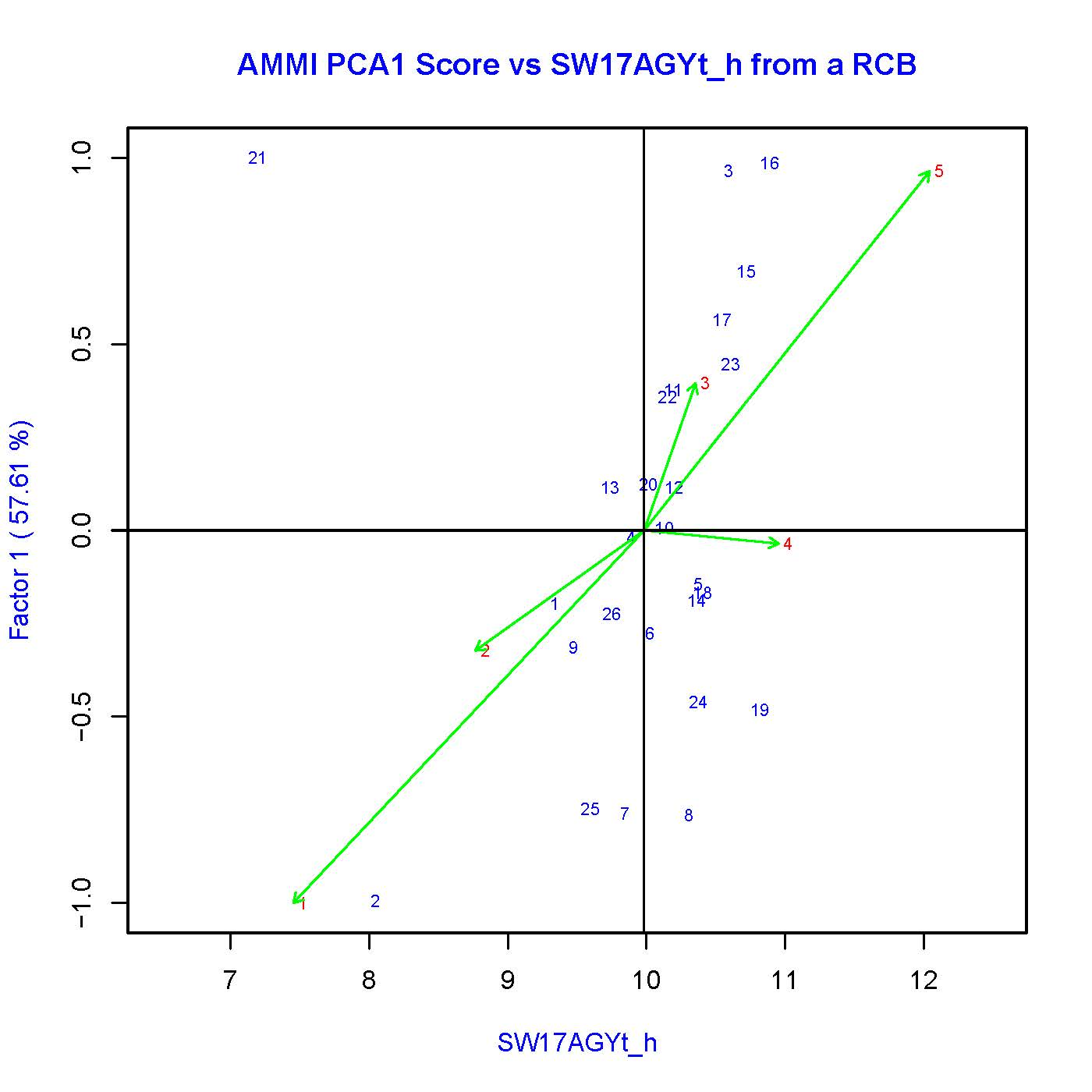


(d) (e) (f)


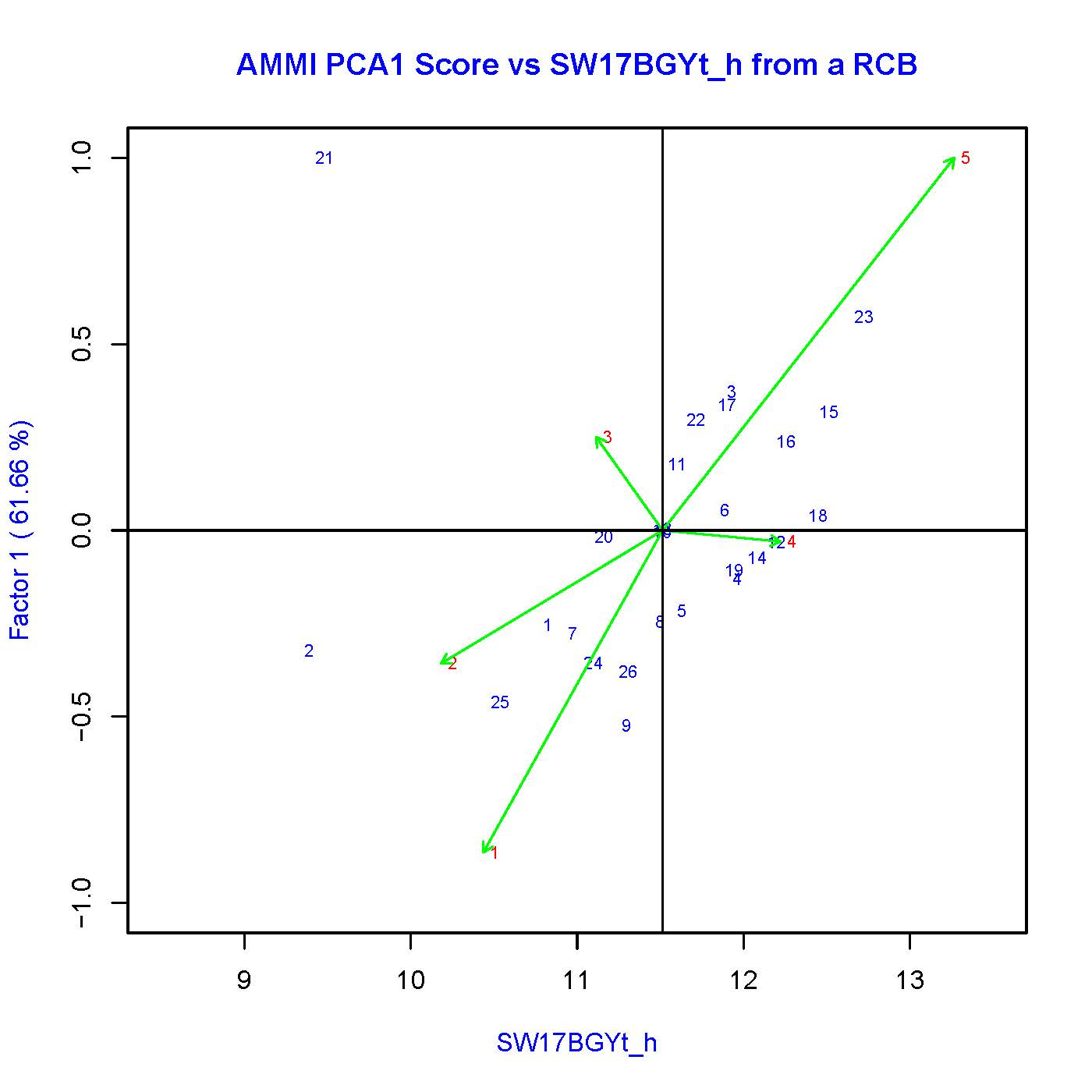

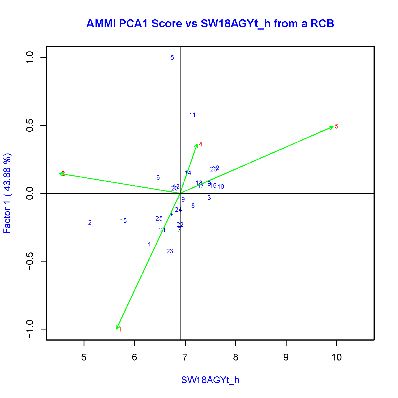

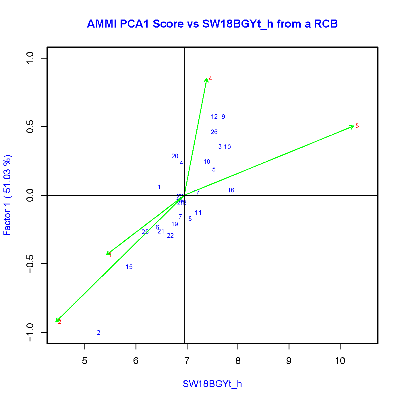


(g) (h) (i)


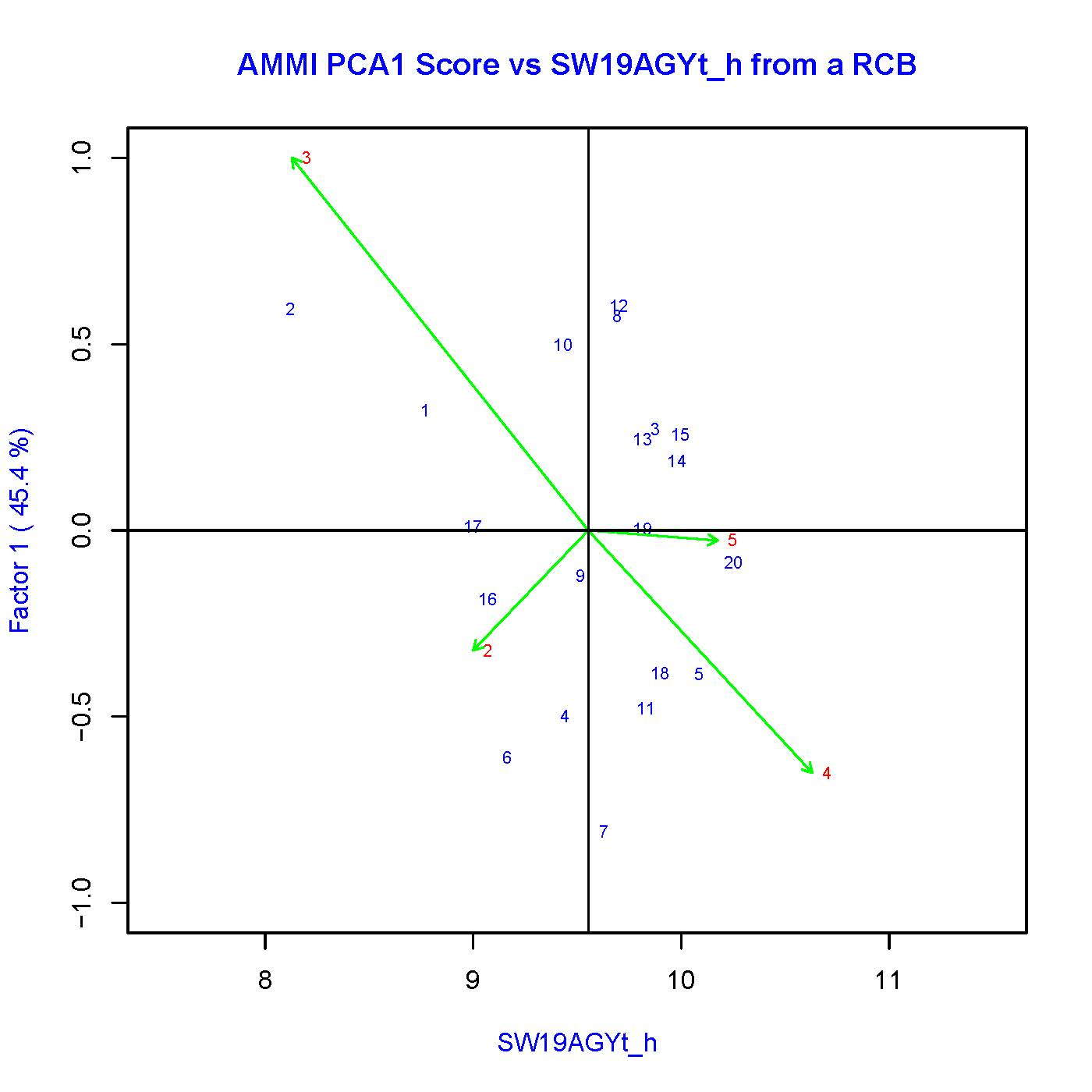

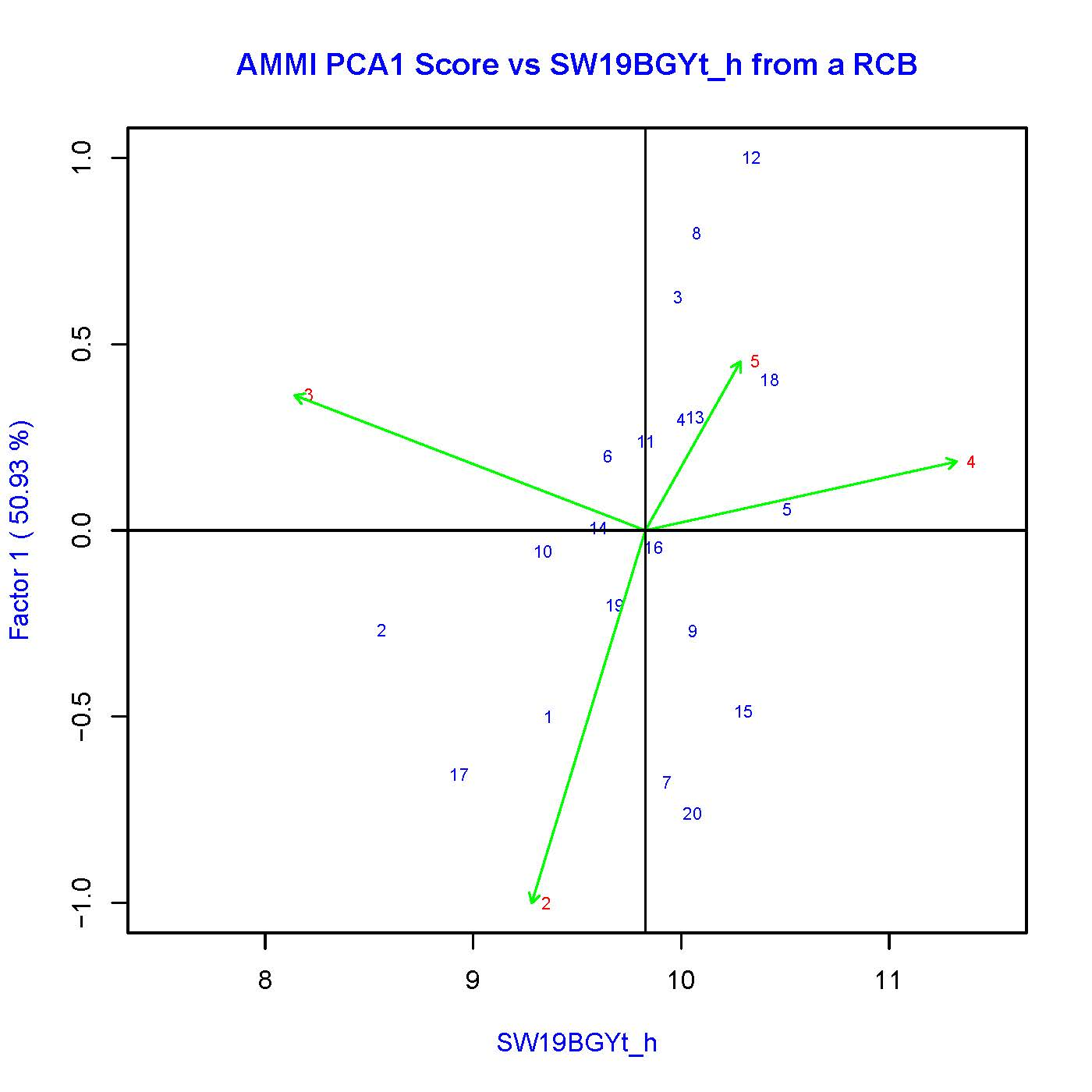

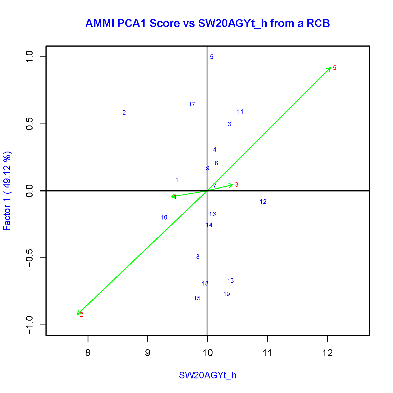


(j)


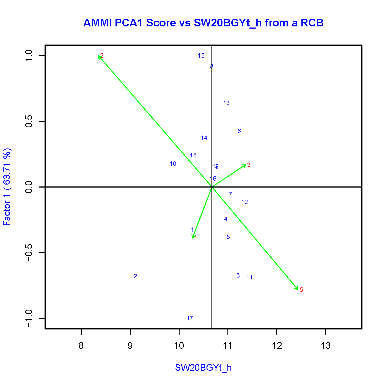


**S Fig 1. Showing AMMI-1 biplot for fungicide untreated (FUT) and fungicide treated (FT) treatment for wheat grain yield for all years. (a) 2016 FUT, (b) 2016 FT, (c) 2017 FUT, (d) 2017 FT, (e) 2018 FUT, (f) 2018 FT, (g) 2019 FUT, (h) 2019 FT, (i) 2020 FUT, (j) 2020 FT.**
